# The Effect of a Tropical Climate on Available Nutrient Resources to Springs in Ophiolite-Hosted, Deep Biosphere Ecosystems in the Philippines

**DOI:** 10.3389/fmicb.2019.00761

**Published:** 2019-05-01

**Authors:** D’Arcy R. Meyer-Dombard, Magdelena R. Osburn, Dawn Cardace, Carlo A. Arcilla

**Affiliations:** ^1^Department of Earth and Environmental Sciences, The University of Illinois at Chicago, Chicago, IL, United States; ^2^Department of Earth and Planetary Sciences, Northwestern University, Evanston, IL, United States; ^3^Department of Geosciences, The University of Rhode Island, Kingston, RI, United States; ^4^Director of Science and Technology-Philippine Nuclear Research Institute, Manilla, Philippines

**Keywords:** serpentinization, carbon isotopes, carbonates, carbon cycling, nitrogen cycling, deep biosphere

## Abstract

Springs hosted in ophiolites are often affected by serpentinization processes. The characteristically low DIC and high CH_4_ and H_2_ gas concentrations of serpentinizing ecosystems have led to interest in hydrogen based metabolisms in these subsurface biomes. However, a true subsurface signature can be difficult to identify in surface expressions such as serpentinizing springs. Here, we explore carbon and nitrogen resources in serpentinization impacted springs in the tropical climate of the Zambales and Palawan ophiolites in the Philippines, with a focus on surface vs. subsurface processes and exogenous vs. endogenous nutrient input. Isotopic signatures in spring fluids, biomass, and carbonates were examined to identify sources and sinks of carbon and nitrogen, carbonate geochemistry, and the effect of seasonal precipitation. Seasonality affected biomass production in both low flow and high flow spring systems. Changes in meteorological precipitation affected δ^13^C_DIC_ and δ^13^C_DOC_ values of the spring fluids, which reflected seasonal gain/loss of atmospheric influence and changes in exogenous DOC input. The primary carbon source in high flow systems was variable, with DOC contributing to biomass in many springs, and a mix of DIC and carbonates contributing to biomass in select locations. However, primary carbon resources in low flow systems may depend more on endogenous than exogenous carbon, even in high precipitation seasons. Isotopic evidence for nitrogen fixation was identified, with seasonal influence only seen in low flow systems. Carbonate formation was found to occur as a mixture of recrystallization/recycling of older carbonates and rapid mineral precipitation (depending on the system), with highly δ^13^C and δ^18^O depleted carbonates occurring in many locations. Subsurface signatures (e.g., low DOC influence on C_biomass_) were most apparent in the driest seasons and lowest flow systems, indicating locations where metabolic processes divorced from surface influences (including hydrogen based metabolisms) are most likely to be occurring.

## Introduction

Recent interest in the terrestrial deep biosphere has been fueled by estimates of biomass (Kallmeyer et al., 2012; Lomstein et al., 2012; McMahon and Parnell, 2014) and reports of unique microbial communities and ecosystem functions (Biddle et al., 2012; Parkes et al., 2014; Solden et al., 2016). The overall impact of the deep biosphere on global biogeochemical cycling remains unknown (Menez et al., 2012), and direct access remains expensive with non-trivial logistics. Surface connected expressions of the terrestrial deep biosphere such as caves, wells, and springs are convenient and less expensive (compared to drilling based endeavors), but elicit questions about authenticity of a subsurface signature. Evidence of subsurface biosphere diversity and function may be overprinted or masked by the oxygenated, photosynthesis-driven, surface biosphere to an unknown degree.

Several studies have attempted to isolate a subsurface signature from terrestrial locations. Arguably the most success has come from studies with direct access to subsurface sampling. These works have relied on comparative metagenomics (e.g., Lau et al., 2014), geochemical modeling integrated with statistical analyses (e.g., Osburn et al., 2014), and innovative culturing techniques (e.g., Rowe et al., 2017) to distinguish subsurface contributions to nutrients, energy, diversity, and biomass. Isolating a subsurface signature when samples are obtained within the surface biome (i.e., springs and seeps) poses additional challenge. More frequently, only individual processes can be linked to the subsurface. For example, the widespread ability to fix nitrogen (Hamilton et al., 2011) or carbon (Osburn et al., 2011; Urschel et al., 2015) using non-photosynthetic pathways in terrestrial hydrothermal systems suggests these traits are maintained due to a general lack of reliable nutrient input from the surface biome.

Stable isotope chemistry of nutrient pools and resident biomass is a useful tool for deep subsurface biosphere applications. Kinetic isotope fractionation associated with biosynthetic machinery discriminates broadly against heavy isotopes, producing ^13^C and ^15^N- depleted biomass relative to sources. Fractionation varies by process for both carbon and nitrogen isotopes and much can be learned or inferred by comparing isotopic ratios of sources and resulting biomass. For example, it has been shown that different carbon fixation pathways fractionate carbon to differing degrees (Berg et al., 2010; Pearson et al., 2016), and the isotopic composition of nitrogen in biomass is affected by how the organism participates in the nitrogen cycle (Brunner et al., 2013; Mobius, 2013; Frey et al., 2014; Zhang et al., 2014). While there is still much to learn concerning the fractionation of carbon or nitrogen by specific groups of organisms under varying environmental conditions, as well as abiotic considerations (McCollom et al., 2010; Li et al., 2012; Wunderlich et al., 2012; Schrenk and Brazelton, 2013; Zhang et al., 2014) a holistic approach of comparing isotopic ratios of bulk carbon, nitrogen, and biomass can reveal broad ecosystem patterns.

This study focuses specifically on surface expressions (springs and seeps) of the deep biosphere sourced in ultramafic rock units in the Philippines. Springs and seeps emanating from ophiolites represent subsurface fluids produced from the process of “serpentinization” that are mixed with fluids of other sources. Briefly, serpentinization is the aqueous alteration of ultramafic rocks (part of the ophiolite body). In terrestrial ophiolites, the process is often recharged by groundwaters, and provides alteration products including mineralogically altered solids, fluids of distinctive geochemistry, gasses such as H_2_ (and possibly CH_4_), and chemical energy abundant enough to fuel chemosynthetic metabolism. Other resources have thoroughly described and reviewed this process and the resulting potential subsurface habitats (McCollom and Bach, 2009; Schrenk and Brazelton, 2013). Serpentinization has been discussed as a possible process fueling life on other planetary bodies (e.g., Mars and icy ocean worlds –Ehlmann et al., 2010; Vance et al., 2016; Deamer and Damer, 2017), as well as a potential platform for the development of life on Earth (Sleep et al., 2011; Russell et al., 2014). In both of these extensions, it is assumed that serpentinization-driven life support is divorced from potential carbon and energy sources supplied from photosynthetic processes. Further, serpentinizing springs and seeps are often noted to have low dissolved organic or inorganic carbon (e.g., Morrill et al., 2013; Szponar et al., 2013). The production of H_2_ gas from the serpentinization process has indicated the potential for hydrogen driven metabolic processes in impacted environments, with an overlying assumption that the presence of hydrogen gas may indicate a more subsurface “signal.” Therefore, separating a true subsurface signal from complicating surface influence at modern, terrestrial, serpentinizing seeps is essential for understanding their utility as a “portal” into the deep subsurface biosphere of modern, ancient, and astrobiological environments.

The isotopic ratios of carbon in total or dissolved inorganic carbon (TIC/DIC) and dissolved organic carbon (DOC) can be compared to that of biomass, allowing conclusions concerning the source of the carbon fueling the biomass production. This approach has been used many times in other terrestrial environments (e.g., Lang et al., 2012; Schubotz et al., 2013; Pearson et al., 2016). There have been several reports from terrestrial serpentinizing seeps and springs that highlight potential carbon and nutrient sources. Both DOC and TIC have been reported in low concentrations in springs located in the Tablelands (Newfoundland, Canada) and The Cedars (CA, United States) areas, with δ^13^C_DOC_ ranging from -22 to -13‰, and δ^13^C_TIC_ ranging from -33 to -2‰ (Brazelton et al., 2013; Morrill et al., 2013; Szponar et al., 2013). Isotopic composition of carbon from both DIC and DOC in the Yanartaş/Chimera site in Turkey are comparable, although concentrations of both are higher than other reported locations (50 and ∼5 ppmC, respectively, Meyer-Dombard et al., 2015). Carbon isotopic compositions of biomass are seldom reported in general for these locations (however, see Meyer-Dombard et al., 2015), but that of the carbonates found in these systems has been widely reported and summarized. The comparison of δ^13^C_carbonate_ and δ^18^O_carbonate_ is useful for determining the sources of carbon and oxygen that formed the carbonates as well as processes involved in that mineral precipitation, and several reports give useful comparisons of carbonates found in serpentinizing seeps from Oman, Costa Rica, Italy, and the western United States (e.g., Szponar et al., 2013; Mervine et al., 2014; Sanchez-Murillo et al., 2014; Falk et al., 2016). The δ^13^C_carbonate_ and δ^18^O_carbonate_ in carbonates from serpentinizing seeps range from ∼-33 to ∼ +3‰ δ^13^C and ∼-20 to ∼ +5‰ δ^18^O. This wide range of isotopic compositions makes it clear that the formation and history of carbonates in serpentinizing systems can follow a varied path, which can sometimes be clarified using “clumped isotope” techniques (e.g., Eiler, 2007).

Among the studied examples of terrestrial serpentinizing seeps, few data are from tropical biomes (Beccaluva et al., 1999; Sanchez-Murillo et al., 2014; Cardace et al., 2015; Woycheese et al., 2015). Tropical surface biomes that deliver significant meteorological precipitation and cover field locations in dense foliage may complicate the isolation of the deep subsurface signal from surficial components. Frequent or heavy precipitation may facilitate the incorporation of exogenous nutrients into the surface exposed seeps and springs, and impact the nutrient availability to the subsurface biosphere. Conversely, addition of meteoric water to the serpentinizing fluids associated with the seeps and springs may locally dilute endogenous nutrient or energy resources, changing the fluid-rock interactions happening in the subsurface. Either scenario could cause fluctuations in sources of energy and carbon between seasons, ultimately affecting the ability to identify true subsurface-driven processes such as hydrogen based metabolisms. While this may incur difficulty in separating a “true” subsurface signal in serpentinizing systems hosted in ophiolites located in tropical biomes, it also provides an opportunity to explore a largely unrecognized exchange between the surface and subsurface biosphere.

Our interest lies in investigating the effect of seasonal meteorological precipitation on the deep subsurface signature from spring locations in the Zambales and Palawan ophiolites in the Philippines. Geologic and geochemical descriptions of both ophiolite exposures and springs have been reported previously (Abrajano et al., 1989; Abrajano and Sturchio, 1990; Cardace et al., 2015; Ilao et al., 2018). Here, we look at carbon isotopic signatures in fluids and solid materials (sediments, biomass, and carbonates), and nitrogen isotopic signatures of biomass, over three precipitation-defined periods. Both the Zambales and Palawan ophiolites are located in Monsoon climatic regimes, with defined wet and dry seasons. In addition, we categorize our sample locations based on the flow rate of subsurface fluids emanating from the springs. The driving questions in this work concern whether increased seasonal precipitation will increase exogenous nutrient input to the surface expressions [springs], or conversely, dilute the available metabolic resources derived from subsurface processes. We hypothesized that seasonal precipitation would differentially impact systems with low flow vs. high flow of subsurface fluids, which is supported by the results given here.

## Materials and Methods

### Description of Field Locations

Both field locations are within the monsoon climate zone of the Philippines. Locations were visited in October 2012 during the beginning of the dry season (193 mm/month average precipitation), September 2013 at the end of the wet season (346 mm/month average precipitation), and January 2017 when the least precipitation is received in our field areas (<20 mm/month average precipitation). Several sites in the Zambales and Palawan ophiolites were sampled, although not all sites were sampled in all three seasons. Table 1 notes the season each location was sampled, and Tables 1, 2 note the depth below fluid surface and distance down the outflow channel, where applicable. In general, both fluids and solids were sampled when they were both accessible/removable. Solids included loose sediments, obvious biofilms, or carbonate features (not all were available at every sample location). Table 2 also provides details on sample name and the type of sample collected. For example, at 10m down the outflow at site ML2, three different solid materials were collected in 2012; “gray sediment,” “carbonate mound,” and “rimstone” (Table 2). Samples with identical names and notations between multiple sample years were taken from the same location, as exactly as possible, based on photographic records of previous sampling efforts. We were unable to sample precipitation during the time that we were in the field.

**Table 1:** Fluid geochemistry including DIC and DOC concentrations and carbon isotopic ratios from 2017 samples during the very dry season.

Sample	Location	Notes	Fluid	Season	Temp.	pH	DIC,	δ^13^C,	DOC,	δ^13^C,
		(outflow	depth		°C		ppmC	DIC ‰	ppmC	DOC ‰
		distance, m)	sampled							
ML1	Manleluag	Cistern pool (0 m)	80 cm	2012, dry	34.36	10.89	0.6	-12.8	0.85	-26.8
				2013, wet	34.35	10.86	0.9	-15.42	0.34	-29.63
		January 26th		2017, v. dry	34.39	11.11	0.2	-14.74	0.1	-23.6
		January 27th		2017, v. dry	34.37	10.69	0.2	-11.23	0.2	-23.9
ML2	Manleluag	Source pool (0 m)	30 cm	2012, dry	34.45	10.85	0.5	-16.5	0.4	-26.0
				2013, wet	34.44	10.83	0.4	-11.0	0.12	-26.0
				2017, v. dry	34.45	10.08	0.4	-17.66	0.6	-28.2
		Spill Pool (1.5 m)	20 cm	2012, dry	34.31	10.85	0.8	-15.0	0.81	-25.5
				2017, v. dry	34.22	10.12	0.3	-14.81	bdl	-20.8
		Outflow (10 m)	10 cm	2012, dry	33.84	10.81	1.5	-18.7	0.5	-27.0
				2017, v. dry	33.51	10.25	nd	nd	0.1	-22.1
		Outflow (18.3 m)	5 cm	2013, wet	32.55	10.23	4.4	-21.13	0.73	-29.14
				2017, v. dry	31.29	10.58	1.7	-20.71	0.7	-27.4
PB1	Poon Bato	Pool 1, main (0 m)	10 cm	2012, dry	31.46	11.27	1.3	-25.4	0.3	-23.0
				2013, wet	30.38	11.25	3.0	-13.0	0.3	-27.0
PB2	Poon Bato	Pool 2, “ice cube”	30 cm	2012, dry	26.76	10.43	6.0	-17.5	1.15	-24.4
		Pool 2, “waterfall”	2 cm	2013, wet	29.68	8.74	22.5	-13.76	1.2	-25.8
PB3	Poon Bato	Pool 3, minor	5 cm	2012, dry	28.58	11.31	nd	nd	0.2	-21.0
PBR	Poon Bato	River^∗^	50 cm	2012, dry	27.88	8.64	18.5	-12.1	0.28	-27.8
		River^∗^	50 cm	2013, wet	26.36	8.3	21.3	-8.15	0.61	-23.55
MF1	Mainit Falls	Source (0 m)	5 cm	2012, dry	40.56	9.68	28.1	-15.3	0.29	-26.4
SS1	San Isidro	Cistern pool (0 m)	147 cm	2017, v. dry	48.0	10.53	0.08	-7.48	bdl	-20.9
GS	Governor’s Sp.	Source (0 m)	15 cm	2017, v. dry	38.83	11.08	0.2	-14.5	bdl	-19.3
		Outflow (5 m)	2 cm	2017, v. dry	38.14	11.13	0.4	-17.4	bdl	-19.5
		Outflow (10.3 m)	2 cm	2017, v. dry	37.8	11.13	0.6	-19.99	2.7	-50.8
NWD	NW Dugout	Main pool (0 m)	30 cm	2017, v. dry	29.27	9.91	2.2	-22.52	1.1	-25.7
DH-4		NWD, well^∗^	200 cm	2017, v. dry	32.78	9.50	10.9	-14.78	7.8	-24.8
PF1	Pinaduguan Falls	‘”Pig” pool (0 m)	10 cm	2017, v. dry	35.66	10.95	0.2	-11.45	bdl	-21.6
PF2		“Apron” pool (0 m)	25 cm	2017, v. dry	35.62	10.8	1.3	-20.83	0.2	-23.7
PFR		River^∗^	50 cm	2017, v. dry	30.0	8.4	81.3	-18.85	1.1	-28.8


*Data for samples during the dry (2012) and wet (2013) seasons have been previously reported (Cardace et al., 2015; Table 2), and are repeated here for seasonal comparison. Carbon isotope ratios expressed as compared to VPDB. nd, not determined; bdl, below detection limit. ^∗^Data for rivers and well are provided as points of reference to the local water table. The rivers are immediately proximal to the sample locations, in both cases. The well “DH-4” was drilled ∼200 m from the NWD pool.*

**Table 2:** Isotopic ratios and wt% of carbon, nitrogen, and oxygen from solid materials (mineral, sediment, biofilm) collected in all three field seasons.

				Inorganic chemistry (carbonates)			Calculated		Organic chemistry (post acidification remnants)				Calculated	
							
Name,	Depth	Notes, sample	year	wt% C,	δ^13^C	δ^18^O	δ^13^C	δ^18^O	wt% C,	δ^13^C	wt% N	δ^15^N	Δ^13^C	Δ^13^C
outflow	below	description^a^		CaCO_3_	(VPDB)‰	(VSMOW)‰	CO_2_(g)^b^‰	CaCO_3_^c^‰	organic	(VPDB)‰		(Air)‰	rel. to	rel. to
distance	fluid surface												DIC	DOC
ML1 0 m	80 cm	Source sedi.	2012	93.93	-17.09	-13.38	-25.94	-9.35	0.74	-28.29	0.061	1.987	-15.51	-1.48
0 m	80 cm	Source sedi.	2013	0.31	-13.58	-12.82	-22.46	nd	0.1	-27.24	0.005	3.096	-11.82	2.38
0 m	80 cm	Source sedi.	2017	13.0	-17.6	-10.8	-26.44	-9.11	0.07	-24.7	0.008	-3.303	-13.49	-0.82
ML2 0 m	30 cm	Source sedi.	2012	0.28	-12.79	-15.53	-21.67	-9.25	0.49	-26.72	0.023	1.17	-10.24	-0.25
1.5 m	20 cm	Spill pool	2012	0.9	-18.84	-18.12	-27.68	-9.25	0.21	-26.57	0.015	2.29	-11.53	-1.03
10 m	10 cm	Gray sedi.	2012	4.76	-19.82	-16.76	-28.70	nd	0.34	-26.52	0.024	1.007	-7.86	0.31
10 m	10 cm	Carb. mound	2012	8.61	-18.87	-15.20	-27.75	nd	0.15	-22.98	0.011	1.911	-4.32*	3.84*
10 m	10 cm	Rimstone	2012	84.06	-19.58	-14.97	-28.45	nd	1.94	-25.01	0.167	0.813	-6.36*	1.80*
0 m	30 cm	Source sedi.	2013	0.21	-12.24	-14.45	-21.46	nd	0.63	-27.50	0.033	1.139	-16.69	-1.99
1.5 m	20 cm	Spill pool	2013	0.14	-14.52	-13.71	nd	nd	0.2	-26.96	0.015	-0.798	-16.2*	-1.45*
10 m	10 cm	Gray sedi.	2013	16.75	-20.48	-18.50	-29.47	nd	0.37	-26.02	0.028	2.001	nd	nd
10 m	10 cm	Rimstone	2013	91.15	-19.66	-15.28	-28.66	nd	14.33	-28.29	1.086	0.463	nd	nd
17.7 m	5 cm	Above apron	2013	61.23	-19.20	-16.83	nd	nd	1.35	-21.40	0.121	1.248	-0.27*	7.74*
18.3 m	5 cm	Apron	2013	108.79	-20.11	-15.87	nd	nd	10.22	-27.10	0.836	0.334	-5.97*	2.04*
19.5 m	5 cm	Below apron	2013	2.68	-19.62	-15.64	-28.62	nd	29.91	-28.03	3.257	-2.231	-6.9	1.11
0 m	30 cm	Source sedi.	2017	2.0	-11.8	-14.9	-20.70	-9.15	0.13	-27.0	0.009	1.494	-9.32	1.22
1.5 m	20 cm	Spill pool	2017	3.0	-19.7	-17.1	-28.52	nd	0.14	-28.0	0.01	0.272	-13.18	-7.19
10 m	10 cm	Gray sedi.	2017	72.0	-19.5	-10.3	-28.42	nd	0.16	-26.9	0.014	-0.214	-8.22*	-4.78*
10 m	10 cm	Rimstone	2017	97.0	-19.5	-11.3	-28.43	nd	0.17	-25.9	0.019	0.139	-7.2*	-3.76*
PB1	10 cm	Main pool	2012	14.0	-20.4	-17.5	-29.48	-8.40	0.13	-27.6	0.009	-1.809	-2.12	-4.63
	2 cm	Minor pool	2012	12.0	-19.1	-15.6	nd	nd	0.11	-28.9	0.009	1.433	-3.49*	-6.00*
	0.25 cm	Terrace	2012	99.5	-25.2	-18.6	nd	nd	0.11	-22.8	0.01	-2.034	2.67*	0.16*
	10 cm	Main pool	2013	103.43	-23.30	-18.30	-32.48	nd	0.35	-25.43	0.025	2.385	-12.63	1.42
	2 cm	Minor pool	2013	68.78	-19.96	-15.46	nd	nd	7.96	-25.56	0.736	-0.314	-12.8*	1.28*
	0.25 cm	Micro-terracette	2013	86.18	-19.68	-13.81	nd	nd	26.57	-25.28	2.632	1.601	-12.5*	1.57*
	0.25 cm	Terrace	2013	99.29	-27.75	-21.89	nd	nd	13.45	-27.14	1.592	-0.395	-14.3*	-0.29*
	2 cm	Muddy pot	2013	59.15	-23.56	-18.99	nd	nd	4.23	-28.09	0.405	-1.137	nd	nd
PB2	25 cm	“Star pool” terraces	2012	92.96	-15.46	-9.37	-25.04	-8.31	6.58	-31.23	0.453	2.101	-13.78	-6.82
	2 cm	“Waterfall”	2012	66.0	-16.5	-10.2	-26.08	-8.31	5.30	-30.3	0.235	0.97	-16.49	-4.45
	30 cm	“Ice cube”	2012	99.0	-14.8	-4.8	-24.40	-8.3	0.17	-30.1	0.009	0.324	-12.6*	-5.65*
	N/A	Litter ref.	2012	28.0	-24.6	-21.10	nd	nd	27.13	-31.4	0.364	0.022	nd	nd
	N/A	Soil ref.	2012	16.0	-14.7	-13.3	nd	nd	0.59	-31.8	0.007	0.685	nd	nd
PB3	5 cm	Main pool, red sedi.	2012	1.07	-18.17	-14.80	-27.36	nd	0.59	-25.80	0.043	2.067	-8.3*	-4.36*
	3 cm	Minor seep, white sedi.	2012	68.14	-13.11	-9.41	-22.60	nd	4.62	-28.48	0.091	2.375	-10.9*	-7.04
MF 0 m	5 cm	Source sedi.	2012	25.0	-10.5	-11.2	-19.64	-6.8	0.11	-26.7	0.016	-3.21	-11.46	-0.35
60 cm	0.5 cm	Outflow	2012	3.0	-13.6	-12.4	nd	nd	0.13	-27.1	0.016	-0.32	-11.8*	-0.69*
2.2 m	0.5 cm	Outflow	2012	99.5	-9.3	-12.5	nd	nd	bdl	-26.9	0.0001	0.492	-11.7*	-0.56*
4.3 m	0.5 cm	Outflow	2012	2.03	-16.29	-15.39	nd	nd	0.30	-27.46	0.031	1.136	-12.2*	-1.07*
PF1	10 cm	“Pig” pool	2017	56.0	-19.8	-11.4	-28.46	-7.21	0.22	-26.5	0.029	6.075	-15.04	-4.89
PF2	25 cm	“Apron” pool	2017	99.5	-13.5	-7.3	-22.23	-7.20	0.05	-29.6	0.003	2.555	-8.73	-5.86
GS 0 m	15 cm	Source exit	2017	17.0	-11.8	-17.4	-20.29	-7.39	0.21	-18.4	0.014	3.892	-3.95	-0.85
0.5 m	10 cm	Spill pool	2017	29.0	-21.7	-17.1	-30.05	-0.03	0.79	-24.2	0.07	3.831	-9.73*	-4.93*
4.5 m	3 cm	Outflow	2017	85.0	-28.1	-24.6	-36.49	-7.21	0.3	-26.2	0.025	2.008	-8.76*	-6.66*
5.0 m	2 cm	Outflow	2017	49.0	-25.6	-20.6	-34.02	-0.02	0.71	-26.1	0.057	2.86	-8.73	-6.63
10.3 m	2 cm	Outflow	2017	74.0	-22.8	-19.9	-31.30	-7.20	0.93	-26.7	0.056	3.306	-6.74	24.07
NWD	30 cm	Source pool carbonate	2017	99.0	-16.5	-10.3	-25.86	-7.20	0.28	-22.3	0.019	-0.675	0.19	3.37
5 m	4 cm	Outflow	2017	99.5	-18.5	-13.5	-27.68	nd	0.17	-22.3	0.009	-0.245	0.17	3.35
12 m	1 cm	Outflow	2017	95.0	-17.1	-11.9	nd	nd	1.71	-20.6	0.042	-1.58	1.92	5.10
12 m	1 cm	Black biofilm	2017	82.0	-11.6	-10.8	nd	nd	11.19	-27.7	0.354	0.769	-5.18	-2.00


*Inorganic chemistry of solids (e.g., carbonates) and organic chemistry are presented separately. In addition, calculated equilibrium carbon enrichment between calcite and CO_*2(g)*_, equilibrium δ^*13*^C CO_*2(g)*_, equilibrium carbonate oxygen isotope composition, and the δ fractionation between organic carbon and DIC and DOC are given. Equilibrium constants can be found in Supplementary Table S1. ^a^Abbreviations in this column are as follows: sedi., sediment; ref., reference; carb., carbonate. ^b^Calculated – equilibrium δ^13^C_CO2(g)_ using ε calcite-CO2(g) and measured δ^13^C_CaCO3_ (Bottinga, 1969). ^c^Calculated – equilibrium carbonate δ18O (O’Neil et al., 1969; Friedman and O’Neil, 1977). ^∗^Estimated using δ^13^C DIC or δ^13^C DOC from a proximal and directly related location.*

**FIGURE 1:**
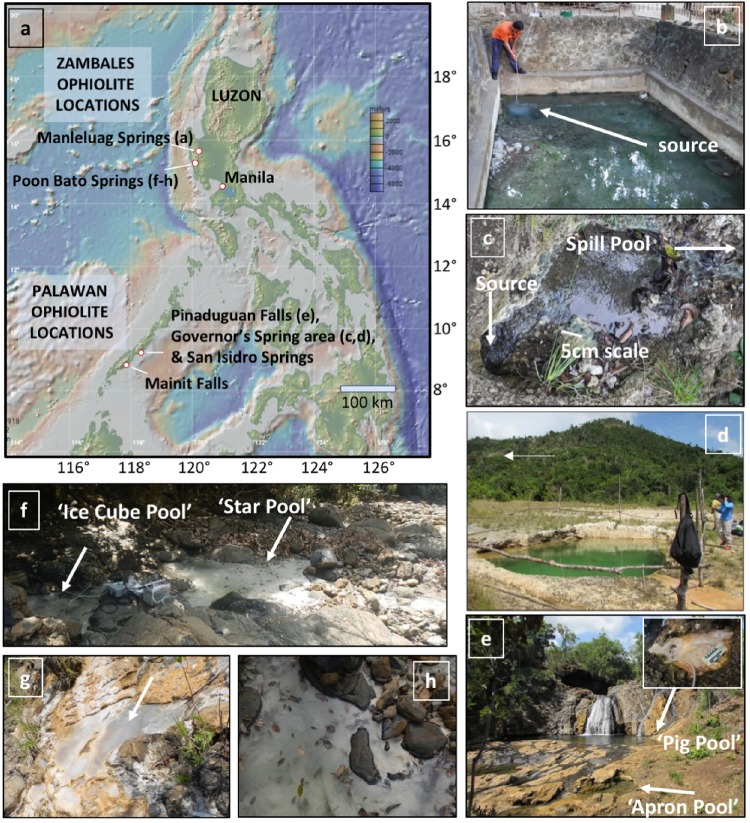
General locations and images of select sample areas. Additional sample area images and site descriptions can be found in Supplementary Figure S1. **(a)** Map of the Philippines with sample regions. Marine Geoscience Data System (MGDS; www.marine-geo.org). **(b)** Cistern pool ML1, Manleluag area. Images of location ML2 and associated outflow sites can be found in Supplementary Figure S1. **(c)** Source pool of “Governor’s Spring.” Small white scale bar noted. White arrow at top right indicates flow direction. **(d)** North West Dugout Pool. White arrow indicates location of spring in **(c)**. **(e)** Pinaduguan Falls. Inset shows close up of “Pig Pool” (PF1). **(f)** Poon Bato “PB2” location, with major features noted. Car battery for scale. **(g)** Poon Bato “PB1” location. Note calcite “cap” on top of the low flow pool (arrow). **(h)** Close up image of “Star Pool” in **(f)**. Note lack of calcite “cap” (bottom of pool is visible).

Images of the sampling areas are provided in Figure 1 with more detailed images and descriptions given in Supplementary Figure S1 for reference. Discharge was estimated by catching runoff into a 500 ml wide mouthed bottle over a 30 s interval, repeated in triplicate and averaged. Where relevant, discharge was measured at multiple points. Sample sites are regarded as being high flow (>2 L/min), with discrete pools and runoff channels, or low flow (<2 L/min) with individual pools or a series of pools and associated carbonate terraces and similar features, but run off channels limited or not present. All locations featuring actively flowing run off channels were estimated to have a discharge rate of >2 L/min. In addition, low flow areas are also designated as “capped” or “uncapped,” where “capped” refers to the presence of a carbonate film on the top of the pool. In such cases, the bottom of the pool is not visible, although the caps may be transient and potentially broken by meteorological precipitation or animal (including human) interaction. Examples of capped and uncapped pools are shown in Figure 1. High flow systems are expected to have an abundance of input from subsurface fluid and gas, and increasing interaction with surface conditions and atmospheric exchange as the fluids progress down the outflow channel. Low flow systems are expected to have slower fluid and gas input from the subsurface, and the degree of interaction with surface conditions depends on the presence of absence of a carbonate cap. We expect that pools with carbonate caps will have more limited atmospheric exchange, as the cap functions as a physical barrier at the surface of the pool.

### Concentration and Isotopic Analysis of Dissolved Inorganic Carbon (DIC) and Dissolved Organic Carbon (DOC) in Spring Fluids

Amber I-CHEM vials and septa were pre-washed and pre-treated as previously described (Cardace et al., 2015). A glass media bottle was used to collect sample from the springs, after being triple rinsed with sample. Once collected, sample was filtered through a Millipore Sterivex GV 0.22 μm filter unit for sterilizing aqueous solutions (Cat. No. SVGVL10RC/Lot No. 1515/00631) into the sample bottle. Some samples required pumping for efficient collection, and a Geotech Environmental Geopump peristaltic pump was used to fill the glass collection bottle. Viton Masterflex tubing (Cole Parmer, Vernon Hills, IL, United States) was used for pumping when collecting DIC samples, at a slow pumping rate of <100 ml/min and as short a pumping distance as possible to minimize gas loss. DOC bottles were filled only after the filter was conditioned with several liters of sample. Both DIC and DOC bottles were filled to the top with sample to exclude air bubbles, and were stored at 4°C.

Samples were analyzed by the University of California, Davis, Stable Isotope Laboratory. Carbon isotopic ratios of carbon in DOC was analyzed with a O.I. Analytical Model 1030 TOC analyzer (Xylem Analytics, College Station, TX, United States), interfaced to a PDZ Europa 20–20 isotope ratio mass spectrometer (Sercon Ltd., Cheshire, United Kingdom) with a GD-100 Gas Trap Interface (Graden Instruments). Several replicates of reference materials were interspersed with samples, including IAEA-600, USGS-40, USGS-41, and Elemental Microanalysis reference materials.

Dissolved inorganic carbon samples were analyzed on a GasBench II system interfaced to a Delta V Plus IRMS (Thermo Fisher Scientific, Bremen, Germany). The fluids were added to a sealed, He-purged exetainer, and acidified to liberate all of the DIC as CO_2_. Reference materials for DIC analyses were lithium carbonate (Acros-1, Acros-2 Li_2_CO_3_, lots measuring δ^13^C -13.4 and -3.85, respectively, Thermo Fisher Scientific, St. Louis, MO, United States) dissolved in degassed deionized water and a deep seawater (both calibrated against NIST 8545). Final δ^13^C values are expressed relative to the international standard VPDB (Vienna PeeDee Belemnite).

### Determination of Endmember Composition and Contributions

An estimation of subsurface endmember δ^13^C_DIC_ was calculated following the method of Miller and Tans (2003), where δ^13^C_DIC_^∗^[DIC] is plotted as a function of [DIC], for each field area, and the slope of the resulting linear regression indicates an estimated δ^13^C_DIC_ for the endmember fluid at that field site. These endmember values of δ^13^C_DIC_ were then used to estimate the fraction of the subsurface endmember remaining following select microbial metabolic processes on the measured δ^13^C_DIC_ pool. Here, a Rayleigh distillation model was used;

$$\delta^{13}C_{observed}=\delta^{13}C_{observed}+10^{3}(\alpha -1)In\left(f\right)$$

Where δ^13^C_observed_ is the measured δ^13^C_DIC_ in the samples, and δ^13^C_source_ is either the estimated δ^13^C_DIC-subsurface_, or δ^13^C_DOC_, as specified below. Fractionation factors, α, were chosen for individual microbial processes. For chemoautotrophic pathways, namely the acetyl Co-A and rTCA cycles, a range of α = 0.964–0.996 as reported in Hayes, 2001 was used. For bacterial photoautotrophy, a range of α = 0.978–0.99 as reported in Hayes, 2001 was used. For hydrogenotrophic methanogenesis, α = 0.945 from carbonate to methane (Waldron et al., 1998) and α = 0.942 from CO_2_ to methane (Krzycki et al., 1987) were used. For each of the above processes utilizing the DIC pool as a carbon source, the δ^13^C_source_ used was the estimated subsurface endmember δ^13^C_DIC_ as calculated for each location as in Miller and Tans, 2003. Processes that produce CO_2_, such as acetoclastic methanogenesis (α = 0.976, Waldron et al., 1998) and methanol methanogenesis (α = 0.932, Rosenfeld and Silvermann, 1959; Silverman and Oyama, 1968) were also considered. For these latter processes, the δ^13^C_source_ used was the measured δ^13^C_DOC_.

### Carbon and Nitrogen Isotopic Ratios in Solid Materials

Solid samples, consisting of sediments, carbonate terrace materials, and any resident microbiota or biofilms, were collected using sterile technique into Whirlpac bags, and kept frozen at -20°C until analysis. Samples were freeze dried, and then ground to a fine powder in glass or agate mortars. Mortars and Pestles were baked at 550°C overnight prior to use. Samples were analyzed in the Osburn Isotope Geobiology Laboratory at Northwestern University.

Carbonate content, assayed as mg CO_2_ and its respective δ^13^C value (hereafter δ^13^C_CO3_) was determined simultaneously via continuous flow on a Thermo Fisher Scientific GasBench II, coupled to a Delta V Plus isotope ratio mass-spectrometer (CF-IRMS) at the Northwestern Stable Isotope Facility. Prior determination of the CO_3_^2-^ content from gravimetric quantification guided the sample amount used for analysis, aiming for ∼10 μmol CO_2_. Samples were weighed into 12 mL Exetainer^®^ vials, which were subsequently septum-sealed and purged with UHP He for 7 min. Approximately 200 μL of 103% H_3_PO_4_ was injected into each vial, and the samples placed into a thermos-stated block at 70°C to allow CO_2_ to evolve overnight. The isotopic composition, δ^13^C_CO3_ is corrected using the periodic sampling of CO_2_ from the H_3_PO_4_-acidified CaCO_3_ standards NBS18 (δ^13^C_V PDB_ = -5.014‰) and NBS19 (δ^13^C_V PDB_ = 1.95‰), and samples reported on the VPDB scale. Estimated precision (1 s.d.) on δ^13^C_CO3_ is ± 0.06‰.

For the determination of wt% organic C and organic N, acidified and decarbonated samples were weighed into tin capsules and combusted online in a Costech 4010 Elemental Analyzer, coupled to a Thermo Fisher Scientific Delta V Plus mass-spectrometer via a ConFloIV. Briefly, ∼10–30 mg of powdered decarbonated samples were weighed, then combusted online in a column containing chromium (III) oxide and silvered cobaltous chloride, held at 980°C. Product gasses were carried over hot Cu reduction column held at 705°C to removed excess O_2_ and convert nitrogen oxides to N_2_. Product CO_2_ and N_2_ were separated by a molecular sieve 5A GC column. The gasses were analyzed via CF-irms, and size corrected. Tank corrections were done by regular calibration against organic standards supplied by Indiana Biogeochemical laboratories (IU acetanilide and IU urea), and placed on the δ^13^C_V PDB_ and δ^15^N_AIR_ scales respectively.

### Carbonate Mineralogy

An Olympus Terra X-ray diffractometer^1^, with the specifications equivalent to the CheMin tool developed for Mars exploration as described in Blake et al. (2012), was used for X-ray diffraction (XRD) analysis. The Terra engages a Co X-ray source and a cooled charge-coupled device (CCD) detector arranged in transmission geometry with the sample, with angular range of 5° to 50° 2θ with <0.35° 2θ resolution (Blake et al., 2012). X-ray tube voltage is typically 30 kV, with a power of 10 W, a step size of 0.05°, and an exposure time of 10 s per step. A minimum of 250 exposures were recorded prior to diffractogram interpretation.

Dry samples were powdered using an agate mortar and pestle, cleaned with isopropyl alcohol between samples. Powder was passed through a standard 150 μm sieve (100-mesh) prior to analysis. Powdered, sieved material was transferred with a spatula to the input hopper of the vibration chamber sample cell, and shaken into the space between two mylar window, to be agitated during analysis, presenting all planes with the mineral sample to the x-ray beam.

The resulting diffractogram was interpreted using XPowder software^2^, which is a commercially available peak search-and-match program that queries the PDF2 database for reference mineral peak information (see text footnote 2). XPowder allows for identification of major minerals; trace minerals can be missed or masked by peaks of other minerals. Diffractograms convey mineral fingerprint information customarily by plotting of diffracted signal intensity on the *y*-axis against °2θ on the *x*-axis. An intensity peak is the result of constructive interference when Bragg’s law (*n*λ = 2*d* sin θ, where n is the “order” of reflection, λ is the incident X-rays wavelength, *d* is spacing between atomic planes in a crystal structure, and θ is the incidence angle) is fulfilled by the incoming x-rays. For reference, for data collected using a Co x-ray tube, the three most prominent *d*-values for minerals of interest are as follows: serpentine (var. lizardite) Mg_3_Si_2_O_5_(OH)_4_, D1: 7.12 Å, D2: 2.379 Å, D3: 3.56 Å; serpentine (var. antigorite) (Mg,Fe++)_3_Si_2_O_5_(OH)_4_, D1: 7.29 Å, D2: 2.53 Å, D3: 3.61 Å; brucite Mg(OH)_2_, D1: 2.365 Å, D2: 4.77 Å, D3: 1.794 Å; hydrotalcite Mg_6_Al_2_(CO_3_)(OH)_16_⋅4(H_2_O), D1: 7.69 Å, D2: 3.88 Å, D3: 2.58 Å; portlandite Ca(OH)_2_, D1: 2.628 Å, D2: 4.9 Å, D3: 1.927 Å; calcite CaCO_3_, D1: 3.035 Å, D2: 2.285 Å, D3: 2.095 Å; magnesite MgCO_3_, D1: 2.742 Å, D2: 2.102 Å, D3: 1.700 Å; artinite Mg_2_(CO_3_)(OH)_2_⋅3(H_2_O), D1: 2.736 Å, D2: 5.34 Å, D3: 3.69 Å; chlorite (var clinochlore) (Mg,Fe++)_5_Al(Si_3_Al)O_10_(OH)_8_, D1: 7.16 Å, D2; 4.77 Å, D3: 3.58 Å; and smectite (var beidellite) Na_0.5_Al_2_(Si_3.5_Al_0.5_)O_10_(OH)_2_⋅n(H_2_O), D1: 2.55 Å, D2: 2.61 Å, D3: 4.52 Å.

In order to interpret co-occurring minerals in association with spring water, an Eh-pH diagram was constructed in Geochemist’s Workbench Act 2. The system was modeled at a temperature of 25°C, at a pressure of 1.013 bars, with log activity HCO_3_^-^ set at -2.699, log activity Ca^2+^ set at 2, log activity Fe^2+^ set at -3, and unit activity of Mg^2+^ and water. The log activity HCO_3_^-^is based on high CO_2_ in to water mixture as low DIC spring water encounters high DIC surface water. The log activity Ca^2+^ set at 2 represents generally observed molalities of [Ca^2+^] near 100 m, which correspond to activities of ∼100, thus log 100 = 2. The log activity Fe^2+^ set at -3 represents generally observed molalities of [Fe^2+^] near 1 mmolal, which correspond to activities of ∼0.001, thus log 0.001 = -3. Unit activity of Mg^2+^ conveys the Ca dominance of the aqueous system, about two orders of magnitude greater than Mg, thus Mg activity taken as one. Unit activity of water is appropriate for most lower salinity, low temperature waters (activity coefficients not impacted by high levels of solutes).

## Results

### Dissolved Carbon (DIC and DOC)

**FIGURE 2:**
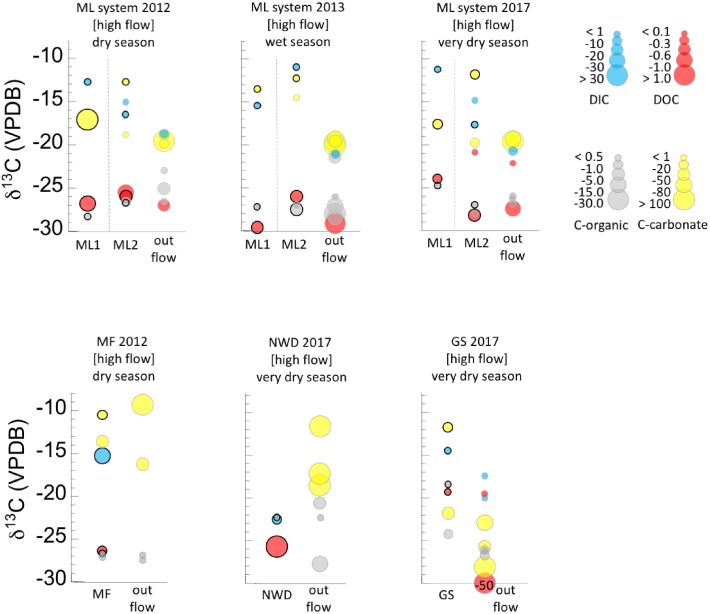
Ranges of concentrations and δ^13^C isotopic composition of dissolved carbon (DIC, DOC), and solid carbon (C-org, C-carbonate) in the high flow systems. Concentration ranges are given by the size of the circle for each value (key at right). Samples are grouped as pool sources plus sample locations within 2 m in one vertical column, and samples farther down the outflow channel are given as a second vertical column. The pool source is distinguished from the other samples by a bold outline around the circle. Refer to Table 1 for full sample names and distances along the outflow. Circles are offset along the horizontal for clarity only. Data are also separated by seasonal sampling, and sample names correspond to the names in the sample location pictures (Figure 1 and Supplementary Figure S1) and Table 1. Dashed lines separate discrete samples within a season. Data can also be found separated by discrete samples in Supplementary Figure S2.

Results of the analysis of DIC and DOC can be found in Table 1. Figure 2, 3 display the carbon isotopic ratios and concentrations of DIC and DOC, separated by flow regime (high flow in Figure 1, low flow in Figure 2) and season. Data are grouped in Figure 2 by samples that are in or near the source pools, vs. those that are part of the extended outflow channel. Calculated estimates of potential subsurface endmember δ^13^C_DIC_ are shown in Supplementary Figure S3, and are ∼-21.8‰ for ML, ∼-22.7‰ for GS, ∼-12.8‰ for NWD, ∼ -13.26‰ for PB, and ∼-22.5‰ for PF locations. The ratio of DOC:DIC at each major field area is considered in Supplementary Figure S4. We are lacking δ^13^C_DIC_ from precipitation during the time periods that we were in the field, so estimates of input from this source of DIC were not considered.

**FIGURE 3:**
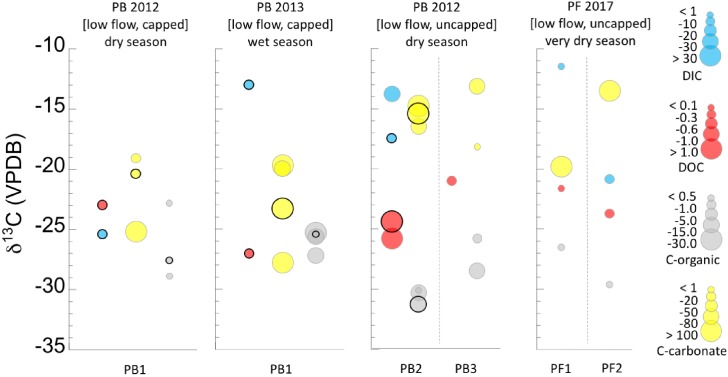
Ranges of concentrations and δ^13^C isotopic compositions of dissolved carbon (DIC, DOC), and solid carbon (C-org, C-carbonate) in the low flow systems. Concentration ranges are given by the size of the circle for each value (key at right). In locations where samples included both a pool source and addition samples in and around the same pool, the pool source is distinguished from the other samples by a bold outline around the circle. Refer to Table 1 for full sample names. Circles are offset along the horizontal for clarity only. Data are also separated by seasonal sampling, and sample names correspond to the names in the sample location pictures (Figure 1 and Supplementary Figure S1) and Table 1. Dashed lines separate discrete samples within a season. Data can also be found separated by discrete samples in Supplementary Figure S2.

#### DIC in High Flow Systems (ML, GS, MF)

General trends in DIC (Figure 2) in high flow systems include low concentrations of DIC (e.g., <1 ppmC) in source pools, continuing downstream to end with an often higher concentration signal (1–10 ppmC). The δ^13^C_DIC_ at the source pools was more enriched relative to downstream locations at all sites and varied between -11 to ∼-18‰ at ML, but ∼-14 to -22‰ in high flow Palawan ophiolite locations. At the high flow system ML2, a sample was taken 1.5 m beyond the mouth of the source pool in 2012 and 2017, and the δ^13^C_DIC_ became briefly more enriched than the source pool across that distance. Discrete source pools associated within a larger spring system [e.g., ML1-ML2 or GS-NWD] had highly variable δ^13^C_DIC_, with as much as a 7–8‰ difference in δ^13^C between them (Figure 2). Precipitation (meteorological) did not influence DIC concentration, but may influence the δ^13^C_DIC_ in the high flow ML systems (the only high flow systems where data from multiple seasons are available). The δ^13^C_DIC_ at ML1 was more ^13^C enriched in drier seasons relative to the wet season, while the δ^13^C_DIC_ at ML2 became more ^13^C depleted in drier seasons relative to the wet season. The highest concentration of DIC in a high flow system was found in MF (dry season). It was concluded previously that this site is influenced in part by non-serpentinizing hydrothermal fluids (Cardace et al., 2015).

Subsurface endmember δ^13^C_DIC_ for sites at ML and GS are depleted by ∼13‰ relative to atmospheric DIC. A Rayleigh distillation model (Supplementary Table S3) predicts a wide range of potential δ^13^C_DIC_ from the subsurface endmember for both areas (∼6–91%) after fractionation by microbial processes that consume DIC, after DIC production and fractionation by acetoclastic and methanol methanogenesis (54–93%). In contrast, the predicted remaining subsurface endmember at NWD is 0% for DIC consuming microbial processes, but 28–86% for acetoclastic and methanol methanogenesis.

#### DIC in Low Flow Systems (PB, PF)

Two DIC datapoints are available for each of the two low flow, uncapped sites (Figure 3). The data range from <1 to ∼ 30 ppmC, and δ^13^C_DIC_ ∼-11 to -21‰. This isotopic signature is comparable to that of the high flow systems, although DIC was more abundant in these lower flow, uncapped pools.

While limited data availability makes it difficult to identify broad patterns, there was a notable difference in the δ^13^C_DIC_ between the wet (∼-13‰) and dry season (∼-25‰) in the low flow, capped systems (Figure 3). This was the most negative δ^13^C_DIC_ found. Concentrations of DIC for both low flow, capped samples was 1–10 ppmC.

The subsurface endmember sources of δ^13^C_DIC_ were predicted to be -13.26‰ to the PB area, and -22.5‰ to the PF area. These calculated endmembers are considered with caution, as they are based on very few data.

#### DOC in High Flow Systems (ML, GS, MF)

DOC concentrations in the high flow systems were highly variable and range from 0 to 2.7 ppmC (Figure 2). Carbon isotopic signatures were typically <-25‰ although several samples taken in the very dry season had more positive values (see ML and GS, Figure 2). Notable outliers to these general trends included a groundwater well associated with site NWD with 7.8 ppmC, and the highly ^13^C depleted DOC (-50.8‰, relative to the DOC at all other locations) found 10.3 m down the outflow of GS. There was little apparent effect of meteorological precipitation on the concentration of DOC in the high flow systems, however, lack of rain may be linked to the most positive δ^13^C_DOC_ values found (-19 to -22‰) which occurred in the very dry season in both ML and GS systems.

#### DOC in Low Flow Systems (PB, PF)

The DOC in the fluids of the low flow, uncapped systems (Figure 3) had a more narrow isotopic range than that in the high flow systems. However, the DOC was ^13^C enriched relative to that of many sites (-21 to -26‰), resembling the range for the very dry season samples at high flow ML and GS. No direct seasonal comparison could be made for these sites, due to lack of data from multiple seasons for identical locations. However, the spring systems sampled in the very dry season (PF) had an order of magnitude less DOC than the spring system sampled in the dry season (PB).

Concentrations of DOC from the low flow, capped systems were between 0.1 and 0.3 ppmC, and the δ^13^C was -23 to -27‰ (Figure 3), more ^13^C depleted than the uncapped, low flow counterparts. In the dry season, DOC was more ^13^C enriched than in the wet season, possibly representing the “leftover” pool in the reservoir, which was not refreshed by new material brought in by meteorological precipitation.

### Geochemistry of Solids/Mineralogy

#### Organic Carbon (Biomass) in High Flow Systems (ML, GS, NWD, MF)

**FIGURE 4:**
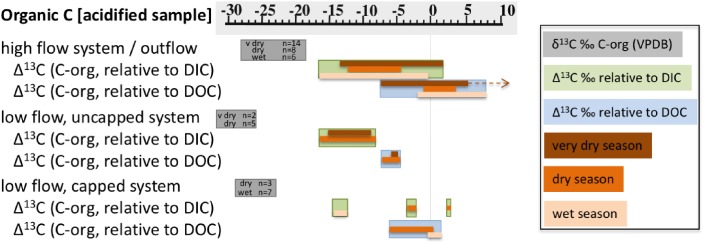
Ranges of measured δ^13^C_organic_ in each of the three types of sample systems (vs. VPDB), and calculated Δ^13^C of organic carbon in solids relative to DIC and DOC. Gray bars note the δ^13^C_organic_ range, and number of analyses per sample type. The sample types are also broken into seasons, key at right. The brown arrow indicates a single outlier data point of ∼ + 24‰.

The carbon isotopic signature of organic carbon in solids (biomass) from high flow systems was in the range of ∼-21 to -28‰, across all seasons and locations (Figure 2 and Table 2). The samples from high flow systems also had the largest range in fractionation between biomass, DIC, and DOC (Figure 4) of all the systems investigated. Biomass in the high flow systems was slightly ^13^C depleted relative to DOC (Table 2 and Figure 2, 4). There were exceptions, usually from samples near the end of the runoff channel which broaden the range of fractionation from DOC. The biomass samples in these systems were the most ^13^C enriched relative to DOC of all the systems considered (Figure 4). In general, the abundance of biomass increased downstream. In addition, biomass was far less abundant in the very dry season, and more abundant in the wet season (Figure 2). The GS system outflow channel presented an atypical case compared to other high flow systems. While the relationship between the biomass and the DOC in the source pool at GS was similar to the other high flow systems, the biomass was as much as 10‰ ^13^C enriched relative to other sites. In addition, the δ^13^C_biomass_ and δ^13^C_DOC_ in the outflow channel of GS were dissimilar, casting doubt that DOC was the source of the carbon for the biomass in the outflow sites.

#### Organic Carbon (Biomass) in Low Flow Systems (PB, PF)

The δ^13^C_biomass_ with the most negative values were found in the low flow, uncapped systems. All samples were >4‰ ^13^C depleted relative to the DOC, and there was very little seasonal variability (Figure 3, 4). These samples also had the most depleted δ^13^C_biomass_ relative to DIC of all the systems sampled, with samples from the very dry season as much as ∼-18‰ depleted relative to DIC (Figure 4). Biomass from the low flow, capped systems had a similar δ^13^C_biomass_ as that from the high flow systems (between ∼-24 to -29‰), with the more negative δ^13^C_biomass_ in samples from the dry season.

#### Carbonates

In the pH range of the spring locations, speciation of DIC is expected to vary as a function of pH; at pH ∼10.3 we can expect about equal proportions of bicarbonate and carbonate components of DIC, below pH = 10.3 bicarbonate will dominate over carbonate, and above pH = 10.3 carbonate is the dominant ion. Fractionation between the two species occurs, but is expected to be small across the temperature and pH ranges studied here. Fractionation as carbonate minerals precipitate will also vary with precipitation rate. Fractionations due to pH and temperature differences between samples/seasons are expected to be minimal, on the order of <2‰ when considering fractionation between H_2_O and CO_2_ due to pH (Halas et al., 1997, Table 4), and <1 to ∼2‰ between bicarbonate and carbonate in our temperature range (Emrich et al., 1970; Table 1). The fractionation due to precipitation rates is also expected to be <1‰ (Turner, 1982). There are no reports that consider all three of these variables on fractionation of DIC or carbonate minerals in the range of conditions of our study sites.

The δ^13^C of solid inorganic carbon (carbonates) were typically only slightly fractionated relative to DIC, showing the most similarity in the wet season and the most fractionation from DIC in the dry and very dry seasons (Table 2 and Figure 2, 3). Again, there were exceptions to this pattern. For example, the carbonates in wet season samples from the PB location (2013), which was low flow and capped, were nearly 15‰ ^13^C depleted relative to the DIC (Figure 3). In these samples, δ^13^C_carbonate_ was more isotopically similar to the δ^13^C_biomass_ and δ^13^C_DOC_. A similar landscape of carbonates ^13^C depleted relative to DIC was found at the GS site, both near the source and farther down the outflow (Figure 2).

**FIGURE 5:**
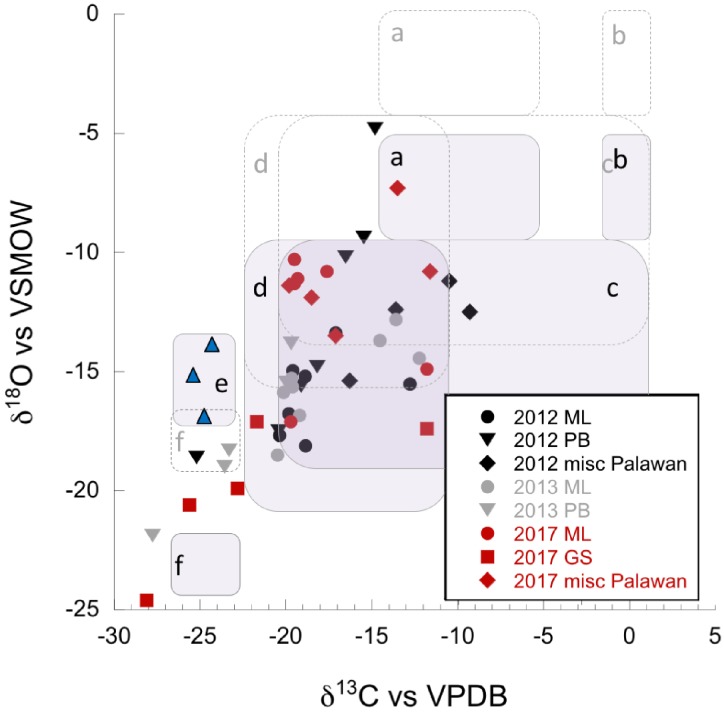
δ^13^C (vs. VPDB), and δ^18^O (vs. VPDB) of carbonates, given in ‰. Black symbols = dry 2012 season, gray symbols = wet 2013 season, and red symbols = very dry 2017 season. Data are found in Table 2. Fields indicate formation and fractionation processes discussed in Clark et al. (1992) and Falk et al. (2016). Dashed boxes indicate the position of fields outlining formation and fractionation processes as discussed in Clark et al. (1992) and Falk et al. (2016) for Oman serpentinization-associated carbonates. Shaded boxes show adjusted positions accounting for the up to ∼5‰ difference in δ^18^O of seasonal and annual rainfall for our study areas. Isotopic data for precipitation were obtained from the Online Isotopes in Precipitation Calculator and are found in Supplementary Table S2 (Bowen and Revenaugh, 2003; Bowen et al., 2005; Bowen, 2019). **(a,b)** These fields represent the expected isotopic ranges for carbonates formed in equilibrium with soil CO_2_, and atmospheric CO_2_, respectively (purposefully no data in the “**b**” fields). **(c)** This field is interpreted by Falk et al. (2016) as containing carbonates that result from mixing of endmember fluids, or recrystallization and/or isotopic exchange in older carbonates. **(d)** Fossil travertine crusts from Oman (Clark et al., 1992), interpreted as shifting toward equilibrium values during secondary recrystallization of more depleted travertine. **(e)** Field containing modern crusts from Oman (Clark et al., 1992), blue triangles, provided for reference. **(f)** Carbonates potentially formed by the CO_2_ hydroxylation process.

Carbonates from the Philippines had some of the most depleted δ^13^C and δ^18^O values reported from terrestrial serpentinizing environments (Table 2 and Figure 5). Values of δ^13^C in our carbonates range from -28.1 to -9.3‰ VPDB, and values of δ^18^O range from -24.6 to -4.8‰ VPDB (5.5–23.4‰ VSMOW). Equilibrium considerations in the formation of carbonates were calculated and are available in Table 2, including a starting δ^13^C_CO2_ and δ^18^O_CO2_. The difference between the measured δ^18^O_carbonate_ and the expected (calculated) δ^18^O_carbonate_ is shown in Figure 7, for samples for which a δ^18^O_water_ was available. In all samples except one (from PB2), the measured δ^18^O_carbonate_ was depleted in ^18^O relative to the calculated, expected value for δ^18^O_carbonate_, with up to -17.39‰ difference. Carbonates depleted in ^18^O relative to equilibrium with water indicate rapid mineral precipitation or precipitation far out of equilibrium.

**FIGURE 6:**
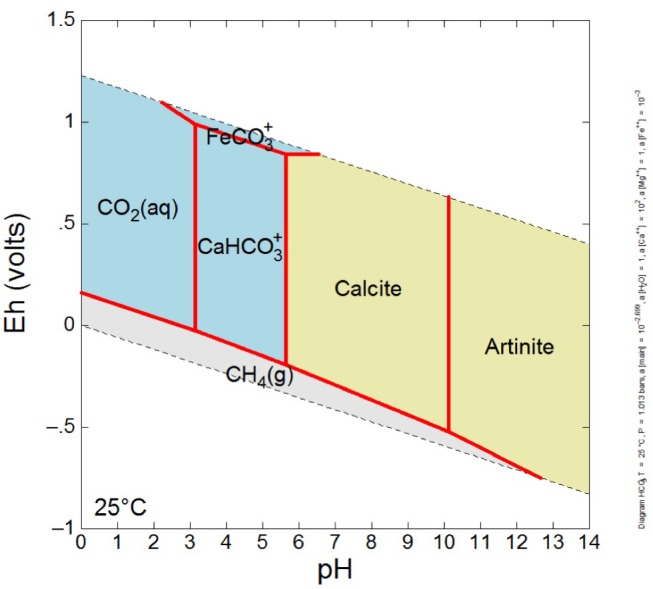
Eh-pH diagram illustrating likely carbonate mineralogy of GS area spring waters, which are Ca-dominated waters with near surface DIC values due to interaction with atmosphere, and bear low concentrations of dissolved Mg and very low concentrations of dissolved Fe. The system was modeled at a temperature of 25°C, at a pressure of 1.013 bars, with log activity HCO_3_^-^ set at –2.699, log activity Ca^2+^ set at 2, log activity Fe^2+^ set at –3, and unit activity of Mg^2+^ and water. Note that artinite is a hydrated magnesium carbonate mineral [Mg_2_(CO_3_)(OH)_2_⋅3H_2_O], and calcite is taken to be pure CaCO_3_. For relevant high pH, low Eh environmental conditions (lower right plotted area), one expects mineral precipitation of artinite, grading to calcite as pH drops (possibly due to organic acid production by microbiology or influx of atmospheric CO_2_).

Because the most negative δ^13^C carbonates were primarily from the GS area, the mineralogy of solid samples from those locations was further explored. The expected carbonate mineralogy is shown in the Eh/pH diagram in Figure 6; measured carbonate mineralogy is given in Figure 8. The model for carbonate stability for a Ca-dominated water with low Mg, low Fe, and near surface DIC values suggests that at the pH and Eh of GS locations, the expected stable magnesium and calcium carbonate minerals are artinite and calcite.

**FIGURE 7:**
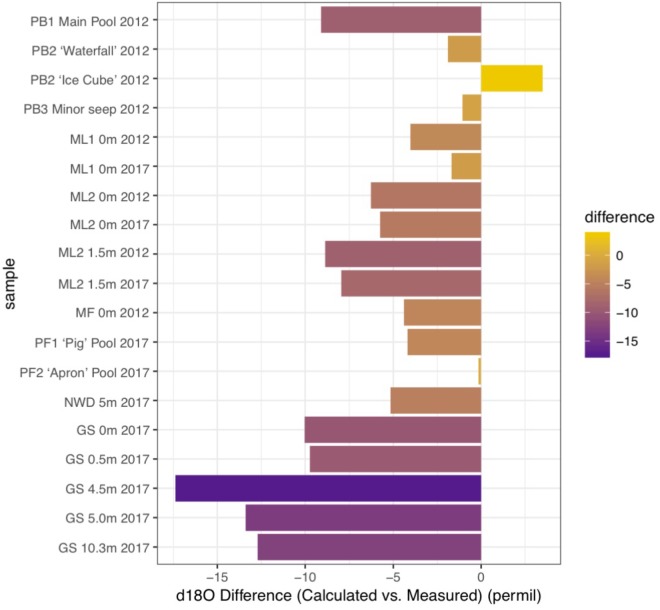
Differences in the values measured for δ^18^O in carbonates and values expected for δ^18^O in carbonates (calculated) in samples from 2012 (dry season) and 2017 (very dry season). Data (see Table 2) are compared for the Poon Bato (PB), Manleluag (ML), Mainit Falls (MF), and San Isidro area springs (PF, NWD, and GS). Positive values are enriched in ^18^O relative to equilibrium, and negative values are depleted relative to equilibrium with water.

**FIGURE 8:**
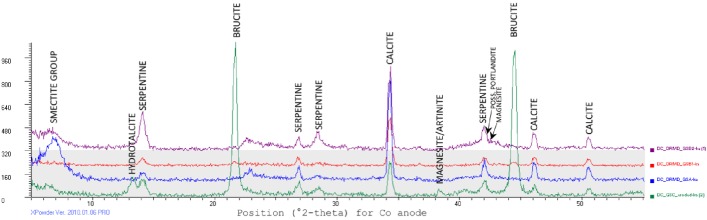
X-ray diffractograms convey mineralogical differences between representative samples of Governor’s Spring solids, collected at spring source (green), two locations along the outflow path (red, purple), and at the base of the main flow channel (blue). Data were collected using an Olympus Terra XRD unit outfitted with Co tube (https://www.olympus-ims.com/en/xrf-xrd/mobile-benchtop-xrd/terra/#!), and peaks were identified using Xpowder (http://www.xpowder.com/). Peaks (intensity on the *y*-axis) indicate strongly diffracted x-rays. Peaks correspond to specific angles (°2-theta, on *x*-axis) at which x-rays are diffracted by specific planes of atoms present in the mineral sample. At spring source (green diffractogram), serpentine peaks co-occur with strong brucite peaks, and associated hydrotalcite. Along outflow path (red and purple diffractograms), carbonate and serpentine minerals dominate, with possible minor brucite, portlandite, and magnesite. The lowest elevation site (blue diffractogram) shows carbonate minerals with a smectite group clay (broad peak, far left).

XRD analysis of GS location carbonates (Figure 8) showed that the outflow sites between 4.5 and 10.3 m are dominated by calcite, serpentinite, clay minerals such as smectite and chlorite, and other smaller proportions of carbonates such as aragonite, magnesite, and artinite, with possible portlandite [Ca(OH)_2_], suggested by variable right-side shoulder near the serpentine peak at ∼42.5 °2-theta. At the source pool alone, brucite [Mg(OH)_2_] and hydrotalcite [Mg_6_Al_2_CO_3_(OH)_16_⋅4H_2_O] are indicated in XRD results, giving evidence for mineral precipitation of hydroxide phases from this OH^-^ dominated spring water where it emerges from the subsurface.

#### Nitrogen

**FIGURE 9:**
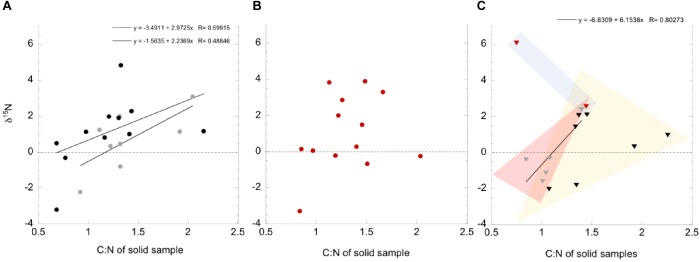
Ranges of measured δ^15^N within organic fraction of solid samples, as a function of the C/N ratio in the samples, divided by 10. Data are found in Table 2. Three sampling seasons are shown; 2013 wet (gray), 2012 dry (black), and 2017 very dry (red) seasons. Circles indicate high flow systems, Triangles indicate low flow systems. The dashed line indicates the expected δ^15^N of atmospheric N_2_. Values below this line are interpreted as produced by fractionation during nitrogen fixation processes, and values above are likely influenced by other nitrogen cycling reactions and nitrogen recycling. **(A)** High flow systems, during the wet and dry seasons. **(B)** High flow systems during the very dry season. **(C)** Low flow systems, all seasons.

The δ^15^N in solid samples (presumably from biomass) compared to the ratio of total carbon to total nitrogen (both as wt.%) are given in Figure 9, broken down by flow system type and season. A relationship between the abundance of nitrogen in biomass and a depleted ^15^N isotopic signature relative to atmospheric may indicate diazotrophic activity, supplying freshly fixed nitrogen to the biomass (e.g., Loiacono et al., 2012 and sources within). In contrast, nitrogen limitation or an ^15^N enriched nitrogen isotopic signature relative to atmospheric may indicate nutrient recycling, exogenous nitrogen addition from eukaryotic surface systems, or microbial nitrogen cycling functions such as nitrification or denitrification. A poorly fit relationship was present between the C:N and δ^15^N in the high flow systems during the seasons with more meteorological precipitation (Figure 9A), but not in the very dry season (Figure 9B). While there were fewer data points available for the lower flow systems (Figure 9C), data cluster according to the season the samples were obtained in. Very dry season samples had the most positive δ^15^N, with high nitrogen biomass (Figure 9C, blue field), dry season samples had the lowest wt% nitrogen in biomass, and δ^15^N data fall between ± 2.3‰ (yellow field), while wet season samples were primarily δ^15^N < 0‰ and low C:N (red field).

## Discussion

Surface derived carbon, such as DOC picked up from plant, animal, or soil sources during overland flow, is characteristically depleted in δ^13^C relative to atmospheric CO_2_. Examples of surface derived carbon can be found in Table 2 – samples from PB1 (“Muddy pot, 2013”) and PB2 (leaf litter and soil reference material, 2012), which range from δ^13^C = -14.7 to -24.6‰. When such biomass is carried into the sample locations after being dissolved or transported as solids, it provides organic carbon with δ^13^C depleted with respect to atmospheric CO_2_. Microbial heterotrophy of organic carbon for biomass production results in very little fractionation of carbon (e.g., Hayes, 1993). In these systems, when the δ^13^C_biomass_ is only barely fractionated with respect to δ^13^C_DOC_ the assumption can be made that DOC was utilized to produce the biomass. Further, it is logical that the source of the DOC is likely primarily from surface environments, rather than produced within the pools, especially during periods of high precipitation. Care needs to be taken when interpreting DOC-biomass relationships in the low precipitation seasons. Measured δ^13^C_DOC_ or δ^13^C_biomass_ values with a large enrichment relative to source DOC are likely products of microbial carbon fixation, a result of recycling of carbon in a closed or semi-closed system, or a mix of heterotrophic and autotrophic growth.

The measured δ^13^C_DIC_ can be the product of multiple processes, including fluid mixing, fractionation following biological activity, and kinetic effects to name a few. Removal of DIC from the source (whether subsurface or surface) by microbial carbon fixation or methanogenesis will change both the concentration of DIC and the δ^13^C_DIC_ in the remaining DIC pool. Some autotrophic organisms are known to fractionate DIC by as much as 36‰ with specific carbon fixation pathways at 25–40°C (Hayes, 2001; House et al., 2003). Likewise, the production of DIC by heterotrophic processes, or acetoclastic methanogenesis will supply DIC to the available pool that is fractionated relative to the source DOC.

Nitrogen fixation fractionates N_2_ only slightly from atmospheric values, and δ^15^N_biomass_ close to 0‰ can be inferred to be a product of nitrogen fixation (Delwiche and Steyn, 1970). Other nitrogen cycle processes produce more negative δ^15^N_biomass_, and values of δ^15^N_biomas_ that are enriched relative to atmospheric N_2_ can result from recycling fixed N_2_ in a closed or partially closed system (e.g., Havig et al., 2011).

The climate of the Philippines affects the primary carbon sources for biomass in serpentinizing spring fluids in the Zambales and Palawan ophiolites. Our goal was to determine the primary carbon source for biomass in both the source pools (where a greater “subsurface” fingerprint might be presumed) and runoff channel locations. Our data indicate that factors that impact the primary carbon source include flow rate of the fluids, and degree of exogenous carbon input from meteorological precipitation-derived DIC and DOC.

Seasonal low meteorological precipitation affects the quantity of biomass present in the sediments at all field locations where multiple seasons were sampled (ML, PB1) – measured biomass was less abundant in the dry and very dry seasons (Figure 2, 3). This decrease in biomass production can not be directly linked to a decrease in DOC concentrations within our sample set. With few exceptions (ML1, and some outflow channel sites of ML2), there is not an apparent decrease of DOC with decreased meteorological precipitation and the highest DOC:DIC ratios are found in very dry season samples (Supplementary Figure S4). However, as discussed below, in a few cases (such as ML during the very dry season) there is evidence of DOC recycling during the drier seasons relative to the wet season. A possible explanation is that biomass does increase in response to an increase in delivery of DOC via overland flow, and the DOC measured during the wet season is “leftover” DOC that has not been consumed. The dynamics of population and metabolic shifts that might be tied to DOC have not been studied previously in these systems.

### High Flow Systems (ML, GS, NWD, MF)

Our results indicate that the primary source of carbon for microbial communities in high flow systems was variable with location and affected by season. Evidence for carbon limitation was found during the very dry season, and the best indication of mixotrophic communities (i.e., indication of both heterotrophic and autotrophic processes) was found at distance down outflow channels where δ^13^C_biomass_ relative to DOC was the most positive (Figure 4). Both DIC and DOC were <1 ppmC in the source pools of most high flow systems (with the exception of MF and NWD). The DOC:DIC of high flow sites was <1 (with only three exceptions), and these represent the highest DOC:DIC ratios found in each sampling season (Supplementary Figure S4).

The carbon isotopic ratio data indicate the primary carbon source incorporated into biomass in the ML1 and ML2 systems was likely DOC. The δ^13^C_biomass_ (Figure 2) in these two springs was only slightly depleted or slightly enriched with respect to DOC (Figure 4); in cases of δ^13^C_biomass_ enrichment relative to DOC, a secondary carbon source of DIC from atmospheric influence or from microbial metabolic byproducts may be invoked. Sampling during the very dry season in the Manleluag area revealed a δ^13^C_DOC_ more enriched than that in other seasons, suggesting carbon recycling in the outflow channel due to lower flow rates and less exogenous carbon delivered by meteorological precipitation, resulting in the residual DOC pool harboring more ^13^C. Modeling of the degree of input from the subsurface δ^13^C_DIC_ was inconclusive with a broad range of incorporation of subsurface source δ^13^C_DIC_ possible (6–93% incorporation). Given that the measured δ^13^C_DIC_ in the ML system is influenced by a wide range of processes we feel the approach used was insufficient to model these dynamics. Our available evidence indicates that carbonate production in the ML systems required a mix of carbon from DIC and atmospheric CO_2_. Carbonates analyzed from the source pools of ML1 and ML2 had enriched δ^18^O and δ^13^C compared to other samples (field “c,” Figure 5). Carbonates in this range of δ^18^O and δ^13^C have been interpreted by others as carbonates formed by remineralization or resulting from mixing of endmember fluids. Farther down the outflow at ML2, carbonates are a mix of “fossil” carbonates and freshly formed carbonates with more depleted δ^13^C and δ^18^O than at the source pools (Figure 5, field “d”).

The other major high flow system, the GS area and associated NWD, was only sampled in the very dry season and the source of carbon for biomass was variable by location. In the source pool of GS, both δ^13^C_DOC_ and δ^13^C_biomass_ were enriched compared to all other sample locations (Figure 2), and the DOC:DIC was the lowest observed (Supplementary Figure S4). The microbial community in the source pool of GS may build biomass from carbon fixation processes, thus producing organic acid byproducts that are ^13^C enriched relative to the DOC or subsurface DIC endmember (Supplementary Figure S3), contributing to the measured δ^13^C_DOC_. Alternatively, the measured δ^13^C_DOC_ may also be a consequence of the very dry season sample time, similar to that observed in the ML systems described above – a comparison with wet season δ^13^C_DOC_ is not available for the GS system. As the fluid at GS flowed downstream, DIC and DOC did not become more abundant (in contrast to the outflow of ML2), and the δ^13^C_biomass_ became more depleted relative to the source pool biomass. Rayleigh modeling of the potential input from a subsurface source of DIC was largely inconclusive for the GS system. DIC at NWD is only slightly enriched relative to DOC. Carbonates produced in and near the source pool at GS occur with brucite [Mg(OH)_2_], and are more ^13^C enriched than other samples (and ∼10‰ ^13^C enriched relative to the next site downstream), possibly representing mixing with endmember fluids or recrystallization of carbonates rather than freshly precipitated carbonates (Figure 5, fields “c, d”). However, Figure 7 shows that the carbonates found in the GS system have the largest difference between the expected δ^18^O and the measured δ^18^O, indicating that rapid carbonate deposition is occurring at this location, even in the source pool. Biomass carbon at the bottom of the outflow channel of GS either incorporated carbon from the carbonates, or influenced the δ^13^C_carbonates_. These carbonates had the most negative ^13^C found in our high flow systems, and there was only <1–4‰ difference between δ^13^C_biomass_ and δ^13^C_carbonate_. This could indicate that carbonate formed quickly in the outflow and microbial waste product DIC was a key source of carbon used to form the carbonate. Another possibility is that the microbial community utilized carbon from the carbonate to build biomass. There is precedent for this latter concept. In high pH serpentinizing systems found at The Cedars (United States), it has been shown that *Serpentimonas* isolates use CaCO_3_ in carbon fixation (Suzuki et al., 2014). Future work will be needed to determine if this is a phenomenon is restricted to the low meteorological precipitation season at GS. Regardless, by 4.5 m down the outflow channel, rapid carbonate deposition has resulted in carbonates with some of the most depleted δ^13^C and δ^18^O relative to other carbonates reported from terrestrial serpentinizing systems.

Evidence for nitrogen fixation in the high flow systems (Figure 9A,B) was limited to the MF site (only sampled in the dry season), the source pool of ML1 in the very dry season, and the distant outflow points of ML2 (but excluding the 2012 dry season samples). These results indicate that the ML system communities have potential for nitrogen fixation under some environmental conditions, and more investigation is needed to determine which members of the community are capable of nitrogen fixation and what conditions enable the process. The MF location is slightly higher temperature than ML, with a likely hydrothermal mixing member (Cardace et al., 2015) and nitrogen fixation processes could be attributed to thermophilic members of the community (e.g., Hamilton et al., 2011; Loiacono et al., 2012).

### Low Flow, Uncapped Systems (PB2, PB3, PF)

While few data are available for low flow systems in general, those data presented in Figure 3 indicate that there may be a relationship between more seasonal meteorological precipitation and an increase in concentrations and volume of DIC, DOC, and biomass in the uncapped systems.

Low flow systems have some of the lowest DOC:DIC ratios of all the samples examined (Supplementary Figure S4), with little variability between seasons. While few data are available for low flow systems, the uncapped pools featured the most depleted δ^13^C_biomass_, relative to DOC, with the largest fractionation from DOC (up to 7‰), regardless of season of sampling. To produce biomass depleted in ^13^C relative to both DOC and DIC, microorganisms either have to use an unidentified, very δ^13^C negative source of carbon, or a large fractionation from DIC needs to occur. These results indicate that even when carbon from surface processes was available in the drier seasons, it is possible that the lower flow, uncapped systems received enough fluid and gas from the subsurface to support a microbial community that engaged in metabolic activities independent from exogenous carbon. During the dry season, these systems may depend on methane or other carbon-bearing gasses sourced from depth. Estimation of the subsurface endmember δ^13^C_DIC_ does not produce results that support this hypothesis. However, it is possible that the estimation of the subsurface source δ^13^C_DIC_ is inaccurate (Supplementary Figure S3) – only a few samples were available for each pool and the Miller-Tans analysis was performed with the PB area samples (capped and uncapped combined) considered as one “site,” rather than separate locations that may have differing endmembers in reality. Alternatively, when exogenous carbon was less abundant in the very dry season, the low flow, uncapped systems may recycle carbon similarly as described above for the high flow systems – metagenomic/metatranscriptomic data could help to clarify the carbon flow for the very dry season.

Regardless of the season of sampling, the nitrogen cycle was dependent on surface-sourced nitrogen as no direct evidence from geochemistry points to active subsurface nitrogen fixation (Figure 9). The low flow, uncapped systems form carbonates slowly and are enriched in δ^13^C and δ^18^O relative to other carbonates sampled, suggesting that they have opportunity to undergo recrystallization as they shift toward equilibrium values (fields “c, d,” Figure 5).

### Low Flow, Capped Systems (PB1)

Seasonality affected the carbon isotopic ratio of DIC and DOC in the “capped” low flow pools, the abundance of biomass present, and possibly the source of carbon for the biomass. In the dry season, the pool fluid is separated from atmospheric and most surface influence by the physical barrier of the carbonate skin on the pool surface (Figure 1g). The low flow, capped systems appear to be forming carbonates at a rapid rate, both on the bottom of the pools and across the surface of the pool. Some of these data fall outside of identified fields in Figure 5, or near field “f,” identified as potentially forming via a CO_2_ hydroxylation process (Falk et al., 2016). Along with the high flow GS locations, these low flow capped carbonates are the most depleted in δ^13^C and δ^18^O of the samples, indicating fresh, fast formation. Under these conditions, new carbon and nutrients can only be obtained from slowly flowing gas and fluid from depth, or from solids already present in the pool. Figure 3 shows how this impacted the DIC, which was more δ^13^C depleted than DOC. The DIC pool was likely influenced by metabolic byproducts from microbial metabolism depleted in ^13^C relative to DIC from surface sources, which were trapped in the fluids under the carbonate cap. The residual DOC pool was also more ^13^C enriched than in the wet season, indicating that heavier δ^13^C_DOC_ was left behind in the DOC pool, non-replenished by surface DOC. The carbon isotopic signature of the biomass was more ^13^C depleted than both DIC and DOC, and could be influenced by incorporation of carbon-bearing gasses from depth, such as in the low flow, uncapped pools. We interpret the wet season δ^13^C_DIC_ as incorporating atmospheric sources, indicating that the cap was at least periodically washed away by precipitation. Biomass in both the wet and dry season carried a nitrogen isotopic signature indicative of nitrogen fixation (Figure 9C and Table 2), in contrast to the uncapped low flow systems. It is unclear why the capacity for nitrogen fixation would be more prevalent in these low flow capped systems than the uncapped systems.

## Conclusion

Our results allow us to broadly characterize the effect of climate and fluid flow on the carbon and nutrient sources of several serpentinization-driven ecosystems in the Zambales and Palawan ophiolites. Increased meteorological precipitation during wetter seasons neither significantly diluted nor added to the DOC and DIC concentrations in the source pools of high flow systems ML1 and ML2, or capped, low flow pool PB1 (the only sample locations where such a direct comparison is possible). Samples farther down outflow channels at ML did have higher concentrations of DOC in the wet season, suggesting that climate may have a larger impact on downstream systems than source pools of high flow systems. However, changes in meteorological precipitation did impact the carbon isotopic ratio of both DIC and DOC in ML and PB1 fluids, which reflected seasonal gain/loss of atmospheric influence on the δ^13^C of DIC, and changes in exogenous DOC input.

The primary carbon source in high flow systems was variable, with DOC contributing more to biomass in the ML system, and a mix of DIC and carbonates contributing to biomass in the GS system. Primary carbon resources in the low flow systems may depend more on endogenous than exogenous carbon. Partially, this may be due to smaller “footprints” of the lower flow systems, affecting the surface area available to receive exogenous materials either washed or dropped into the systems. Carbonate “caps” on the very lowest flow systems seasonally isolate the pools from both organic and inorganic exogenous carbon.

The search for a true “subsurface” signature in the Zambales and Palawan serpentinizing systems has concluded that the highest degree of subsurface influence is found in the low flow systems, and in select source pools of the high flow systems (namely GS). Rapid mineral precipitation of carbonate, and δ^13^C_biomass_ that was depleted relative to δ^13^C_DOC_ highlights potential true subsurface signals. Biomass can not be produced from surface derived δ^13^C_DOC_ with a resulting depleted δ^13^C relative to that source – therefore a subsurface process can be assumed. The drier the climate, the more the subsurface carbon signature is apparent, making it more likely that processes such as hydrogen based metabolisms (methanogenesis or sulfate reduction, for example) are key in ecosystem functioning. In the very dry season, evidence for ^13^C_DOC_ pool enrichment relative to source DOC, without subsequent ^13^C_biomass_ enrichment indicates that more autotrophy and/or methane-driven (and hydrogen dependent) metabolic schemes were in action while DOC was limited.

Future sampling will focus on obtaining multi-season samples from all these locations, with endmember sampling, to further explore the validity of this conclusion. Higher flow systems at ML should be considered very carefully with respect to subsurface signatures, and the degree of surface impact on the geochemistry and microbiology. Previous work identified relict subsurface genetic capacity in the ML systems, and future work will focus on separating surface vs. subsurface function in microbial systems, with an eye to identifying populations that are actively using hydrogen-driven vs. C_organic_-driven metabolic processes. High flow GS and lower flowing capped systems, including PB1 should be the focus of future subsurface biosphere investigations. Further consideration of carbon and nitrogen cycling potential will include insight from metagenomic datasets. These will clarify the presence/absence of the genetic capacity for the microbial communities found in these locations to participate in carbon and nitrogen cycling, identify metabolisms that use the abundant hydrogen present in these systems, and allow deeper interpretation of these geochemical data presented here.

## Author Contributions

All authors contributed to the data collection and interpretation in this manuscript.

## Conflict of Interest Statement

The authors declare that the research was conducted in the absence of any commercial or financial relationships that could be construed as a potential conflict of interest.

## Footnotes

1 https://www.olympus-ims.com/en/xrf-xrd/mobile-benchtop-xrd/terra/#!

2 http://www.xpowder.com/
