# Supplementary material for: The Effect of a Tropical Climate on Available Nutrient Resources to Springs in Ophiolite-Hosted, Deep Biosphere Ecosystems in the Philippines
Source: Front Microbiol. 2019 May 1;10:761. doi: 10.3389/fmicb.2019.00761 (PMC6504838; doi:10.3389/fmicb.2019.00761)
Supplement: Supplementary file 1 [file Table_1.pdf]

Supplemental Table 1. Calculated equilibrium constants for values reported in manuscript Table 2. [a] Equilibrium constants used in calculation of equilibrium carbon enrichment between calcite and $CO_{2(g)}$, equilibrium $\delta^{13}C$ $CO_{2(g)}$. [b] Equilibrium carbonate oxygen isotope composition.

Name, outflow distance	Depth below fluid surface	Notes, sample description	year	equilibrium constant $\delta^{13}C$ ‰ [a]	equilibrium constant $\delta^{18}O$ ‰ [b]
**ML1 0m**	30 cm	Source sedi.	2012	1.00909	1.00005
**0m**	30 cm	Source sedi.	2013	1.00909	1.00005
**0m**	30 cm	Source sedi.	2017	1.00909	1.00005
**ML2 0m**	20 cm	Source sedi.	2012	1.00908	1.00005
**1.5m**	10 cm	Spill pool	2012	1.00909	1.00005
**10m**	10 cm	Grey sedi.	2012	1.00914	1.00006
**10m**	10 cm	Carb. mound	2012	1.00914	NA
**10m**	10 cm	Rimstone	2012	1.00914	NA
**0m**	20 cm	Source sedi.	2013	1.00908	1.00005
**1.5m**	10 cm	Spill pool	2013	NA	NA
**10m**	10 cm	Grey sedi.	2013	1.00927	1.00008
**10m**	10 cm	Rimstone	2013	1.00927	NA
**17.7m**	5 cm	Above apron	2013	NA	NA
**18.3m**	5 cm	Apron	2013	NA	NA
**19.5m**	5 cm	Below apron	2013	1.00927	1.00008
**0m**	20 cm	Source sedi.	2017	1.00908	1.00005
**1.5m**	10 cm	Spill pool	2017	1.00910	1.00005
**10m**	10 cm	Grey sedi.	2017	1.00917	1.00007
**10m**	10 cm	Rimstone	2017	1.00917	NA
**PB1**	10 cm	Main pool	2012	1.00938	1.00011
	2 cm	Minor pool	2012	NA	NA
	0.25 cm	Terrace	2012	NA	NA
	10 cm	Main pool	2013	1.00949	1.00013
	2 cm	Minor pool	2013	NA	NA
	0.25 cm	Micro-terracette	2013	NA	NA
	0.25 cm	Terrace	2013	NA	NA
	2 cm	Muddy pot	2013	NA	NA
**PB2**	15 cm	‘Star Pool’ terraces	2012	1.00982	1.00019
	2 cm	‘Waterfall’	2012	1.00984	1.00019
	20 cm	‘Ice cube’	2012	1.00987	1.00020
	N/A	Litter ref.	2012	NA	NA
	N/A	Soil ref.	2012	NA	NA
**PB3**	5 cm	Main pool, red sedi.	2012	1.00945	1.00012
	3 cm	Minor seep, white sedi.	2012	1.00968	1.00016
**MF 0m**	5 cm	Source sedi.	2012	1.00933	1.00010
**60cm**	0.5 cm	Outflow	2012	NA	NA
**2.2m**	0.5 cm	Outflow	2012	NA	NA
**4.3m**	0.5 cm	Outflow	2012	NA	NA
**PF1**	5 cm	‘Pig’ pool	2017	1.00896	1.00003
**PF2**	15 cm	‘Apron’ pool	2017	1.00896	1.00003

**GS 0m**	10 cm	Source exit	2017	1.00864	0.99996
**0.5m**	10 cm	Spill pool	2017	1.00865	0.99997
**4.5m**	3 cm	Outflow	2017	1.00870	0.99998
**5.0m**	2 cm	Outflow	2017	1.00872	0.99998
**10.3m**	2 cm	Outflow	2017	1.00875	0.99999
**NWD**	10 cm	Source pool carbonate	2017	1.00961	1.00015
**5m**	4 cm	Outflow	2017	1.00941	1.00011
**12m**	1 cm	Outflow	2017	NA	NA
**12m**	1 cm	Black biofilm	2017	NA	NA
