# Supplementary material for: The Effect of a Tropical Climate on Available Nutrient Resources to Springs in Ophiolite-Hosted, Deep Biosphere Ecosystems in the Philippines
Source: Front Microbiol. 2019 May 1;10:761. doi: 10.3389/fmicb.2019.00761 (PMC6504838; doi:10.3389/fmicb.2019.00761)
Supplement: Supplementary file 3 [file Table_3.pdf]

**Supplemental Table 3**. Estimations of the contributions to the measured $\delta^{13}C_{DIC}$ from the calculated subsurface DIC endmember (after DIC consuming or producing microbial processes fractionate the DIC endmember). The first three data columns, which include DIC consuming processes, are summarized in the 'combined' column.

| **Sample** | **season** | **CA[a]** | **PA[b]** | **HM[c]** | **Combined** | **AM, MM[d]** |
|---|---|---|---|---|---|---|
| **ML1, 0m** | 2012, dry | 10-78% | 40-66% | 84-85% | 10-85% | 55-81% |
| | 2013, wet | 20-84% | 53-75% | 89-90% | 20-90% | 55-81% |
| | 2017, v.dry | 17-82% | 49-72% | 88% | 17-88% | 69-88% |
| **ML2, 0m** | 2012, dry | 26-86% | 58-78% | 90-91% | 26-91% | 66-86% |
| | 2013, wet | 6-74% | 33-61% | 82-83% | 6-83% | 54-81% |
| | 2017, v.dry | 35-89% | 66-83% | 93% | 35-93% | 64-86% |
| **ML2, 1.5m** | 2012, dry | 18-83% | 51-73% | 88-89% | 18-89% | 65-86% |
| | 2017, v.dry | 17-82% | 49-73% | 88-89% | 17-89% | 78-92% |
| **PB1, 0m** | 2012, dry | 0% | 0% | 0% | 0% | 0% |
| | 2013, wet | 89-99% | 96-98% | 99% | 89-99% | 56-81% |
| **PB2, 0m** | 2012, dry | 0% | 0% | 0% | 0% | 9-75% |
| **GS, 0m** | 2017, v.dry | 13-80% | 44-69% | 86-87% | 13-87% | 82-93% |
| **NWD, 0m** | 2017, v.dry | 0% | 0% | 0% | 0% | 28-64% |
| **PF1, 0m** | 2017, v.dry | 63-74% | 33-61% | 82-83% | 63-83% | 66-86% |
| **PF2, 0m** | 2017, v.dry | 65-95% | 84-93% | 97% | 65-97% | 89-96% |

*** Estimated from Rayleigh distillation;

$$\delta^{13}C_{observed} = \delta^{13}C_{source} + 10^3(\alpha - 1)\ln(f)$$

Where $\delta^{13}C_{observed}$ here is the measured $\delta^{13}C_{DIC}$ in the samples, and $\delta^{13}C_{source}$ is either the estimated $\delta^{13}C_{DIC\text{-}subsurface}$, or $\delta^{13}C_{DOC}$, as specified in notes below. Fractionation factor, $\alpha$ , was chosen for individual microbial processes, as noted below.

[a] CA = chemoautotrophic pathways, namely acetyl Co-A and rTCA cycles. Range of $\alpha$ = 0.964-0.996 as reported in Hayes, 2001. The $\delta^{13}C_{source}$ used was the estimated $\delta^{13}C_{DIC\text{-}subsurface}$ as calculated for each location as in Miller & Tans, 2003. See supplemental figure 3 for values of $\delta^{13}C_{DIC\text{-}subsurface}$.

[b] PA = photoautotrophy (bacterial). Range of $\alpha$ = 0.978-0.99 as reported in Hayes, 2001. The $\delta^{13}C_{source}$ used was the estimated $\delta^{13}C_{DIC\text{-}subsurface}$ as calculated for each location as in Miller & Tans, 2003. See supplemental figure 3 for values of $\delta^{13}C_{DIC\text{-}subsurface}$.

[c] HM = hydrogenotrophic methanogenesis. $\alpha$ = 0.945 from carbonate (Waldron et al., 1998) and $\alpha$ = 0.942 from CO2 (Krzycki et al., 1987). The $\delta^{13}C_{source}$ used was the estimated $\delta^{13}C_{DIC\text{-}subsurface}$

as calculated for each location as in Miller & Tans, 2003. See supplemental figure 3 for values of $\delta^{13}C_{DIC\text{-}subsurface}$.

[d] AM = acetoclastic methanogenesis, $\alpha$ = 0.976 (Waldron et al., 1998);  MM = methanol methanogenesis, $\alpha$ = 0.932 (Silverman et al., 1959). The $\delta^{13}C_{source}$ used was the measured $\delta^{13}C_{DOC}$.
