# Supplementary material for: The Effect of a Tropical Climate on Available Nutrient Resources to Springs in Ophiolite-Hosted, Deep Biosphere Ecosystems in the Philippines
Source: Front Microbiol. 2019 May 1;10:761. doi: 10.3389/fmicb.2019.00761 (PMC6504838; doi:10.3389/fmicb.2019.00761)
Supplement: Supplementary file 4 [file Image_1.pdf]

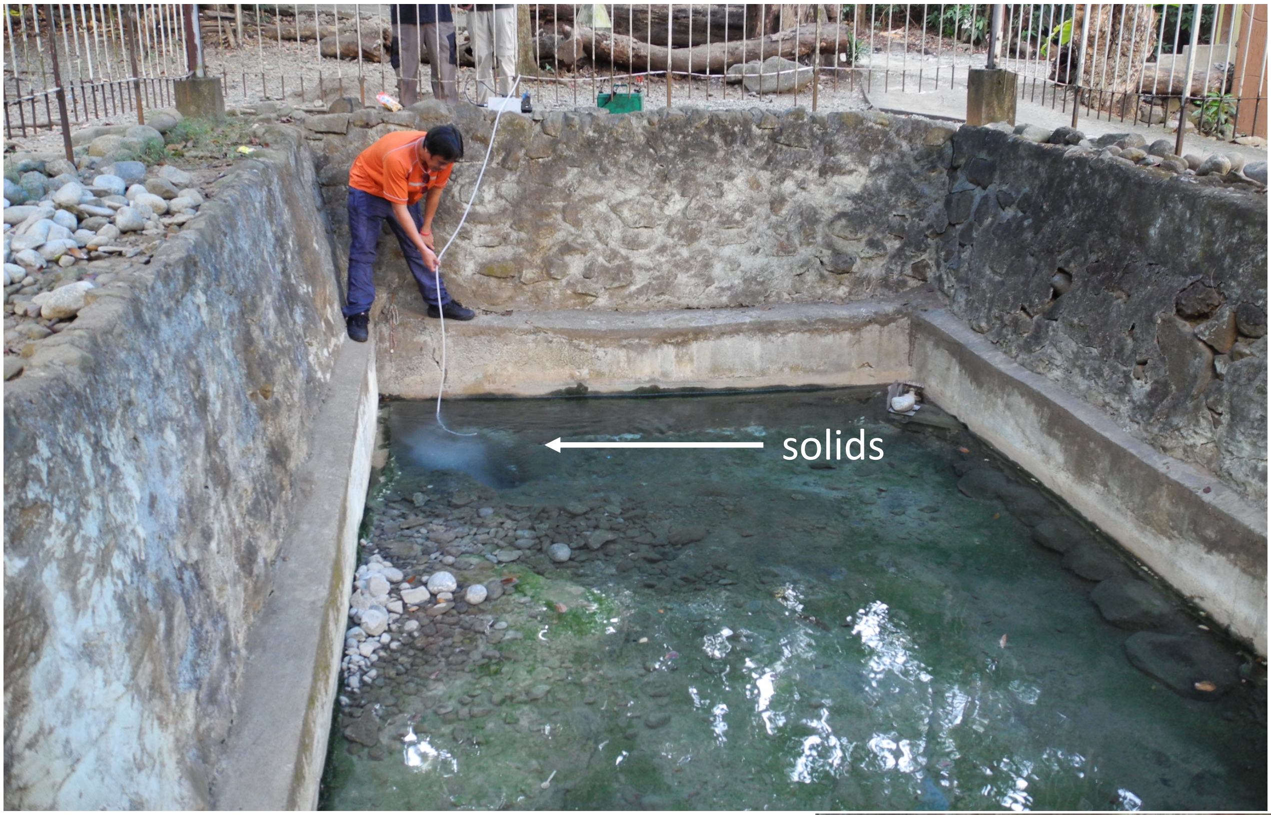

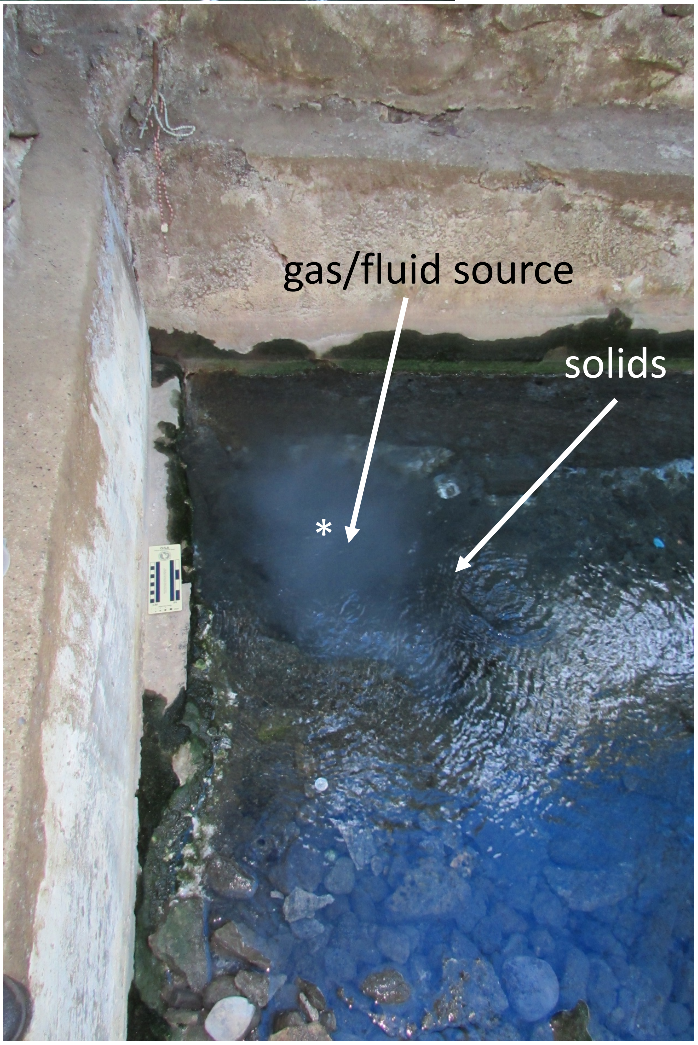

a] Manleluag Spring #1 (ML1). Cistern Pool - a natural spring surrounded by a cistern. The spring source (0m) and associated sediments were sampled in all three seasons (2012, 2013, and 2017). Asterisk marks location of fluid sampling.

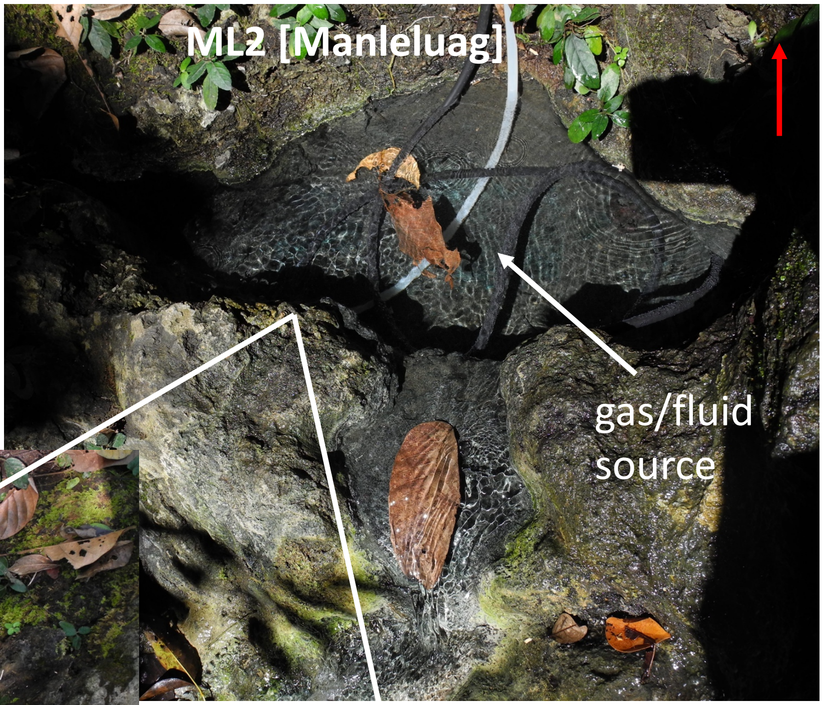

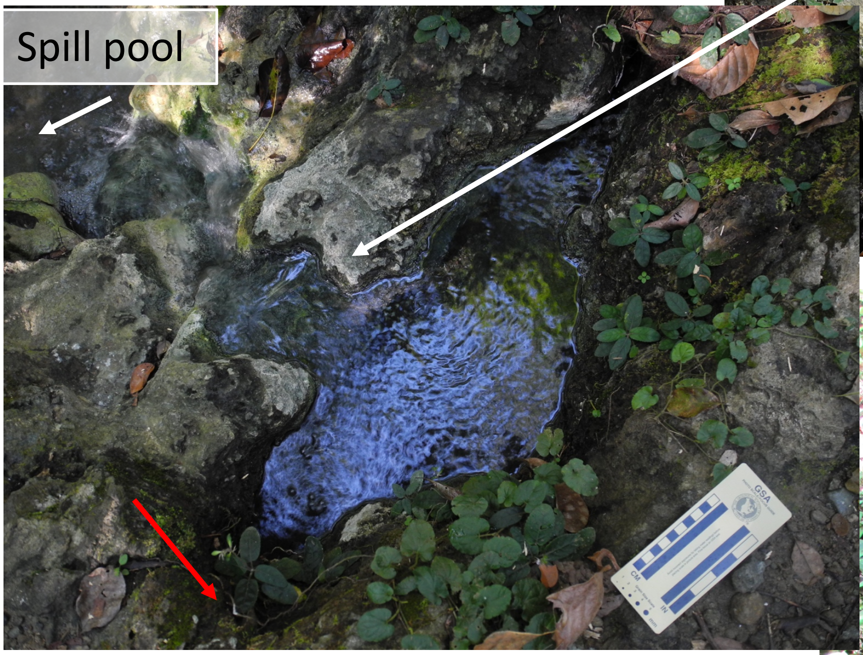

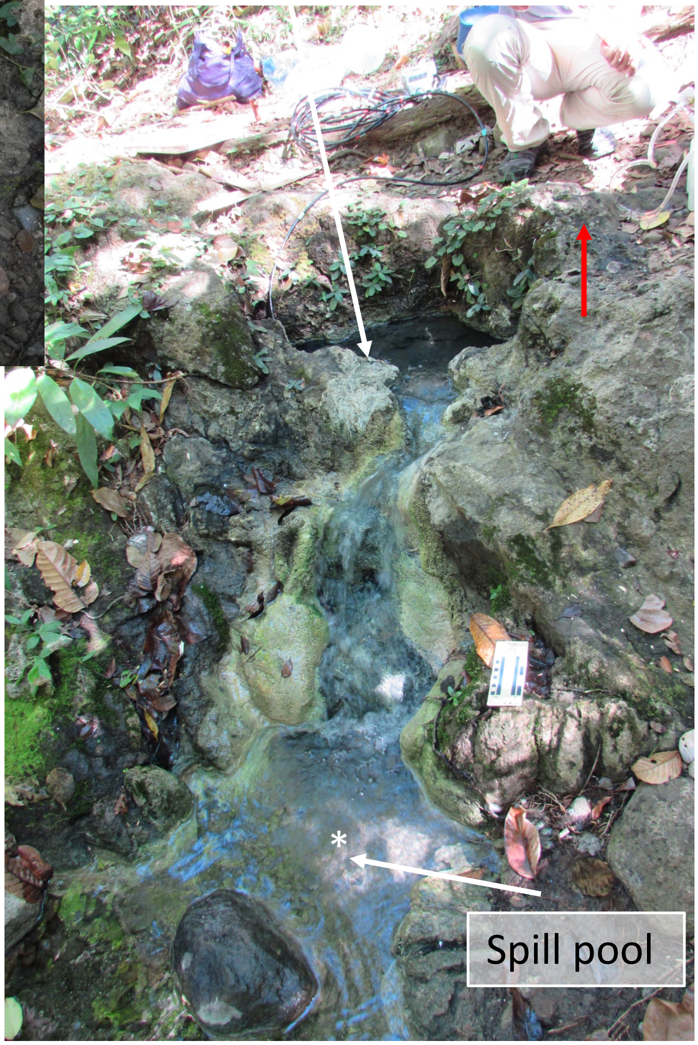

b] Manleluag Spring #2 (ML2). A natural spring seated in the vegetation. Images show the source pool. Red arrows indicate image orientation to North. Large white arrows connect the same location at the lip of the source pool. The spill pool is located 1.5m downstream of the source. Both the spring source and the spill pool were sampled in all three seasons (2012, 2013, and 2017). Asterisk marks location of fluid sampling.

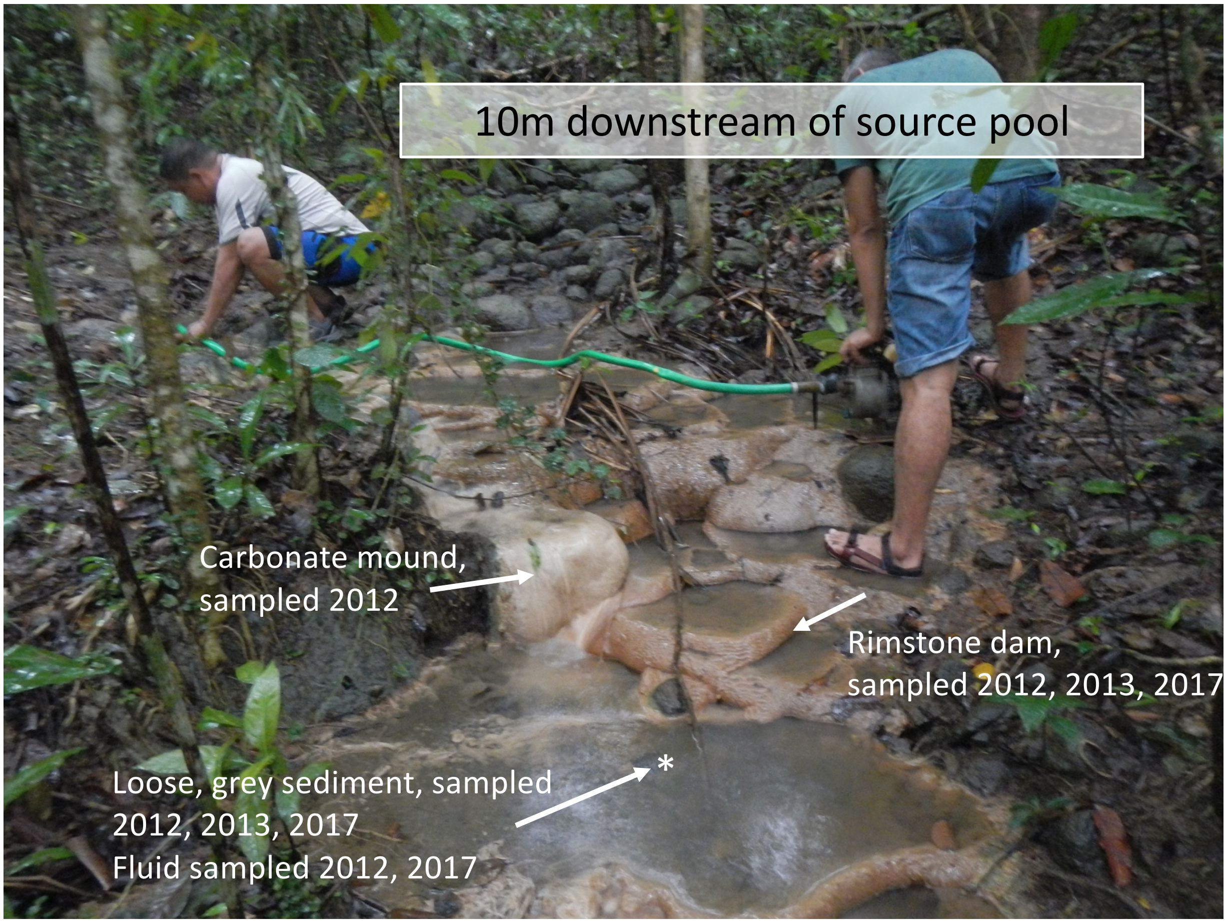

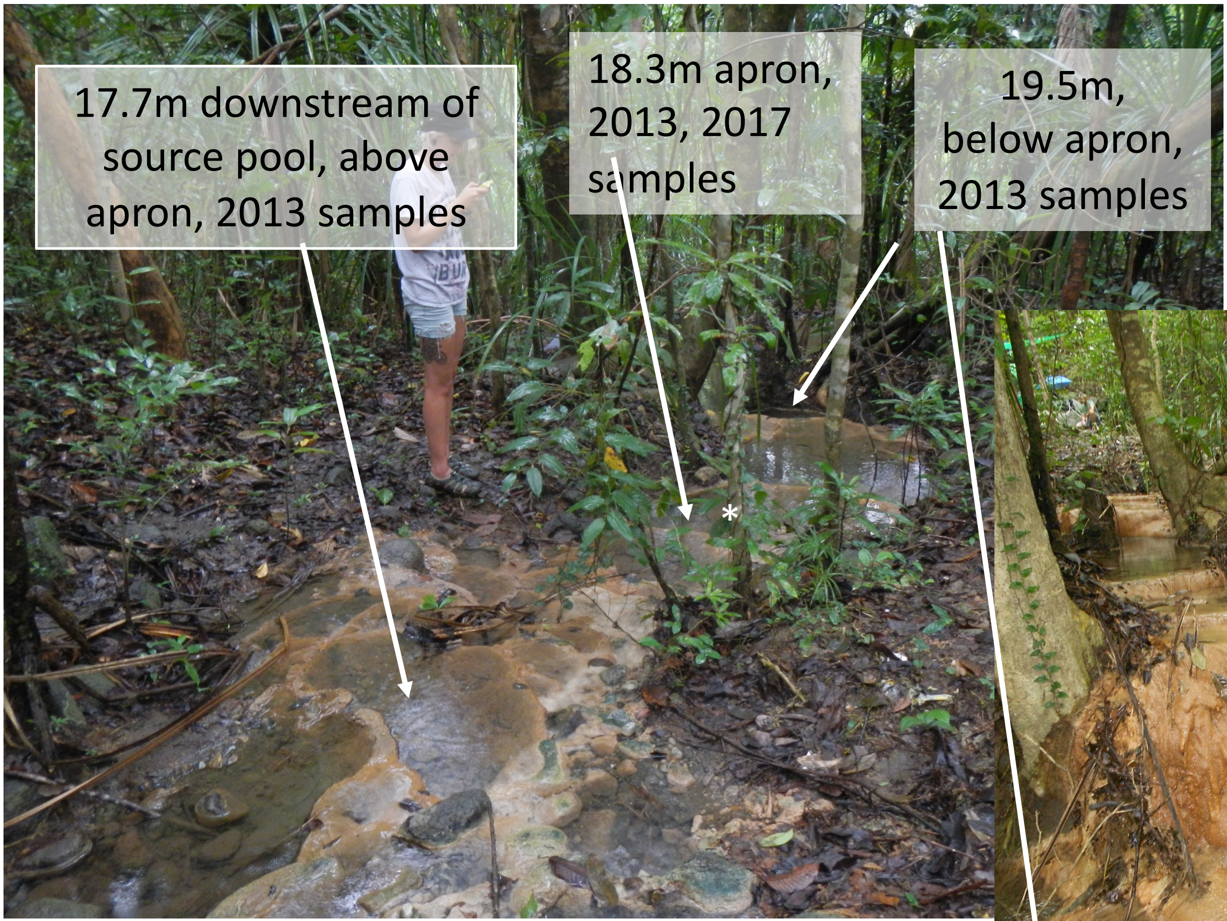

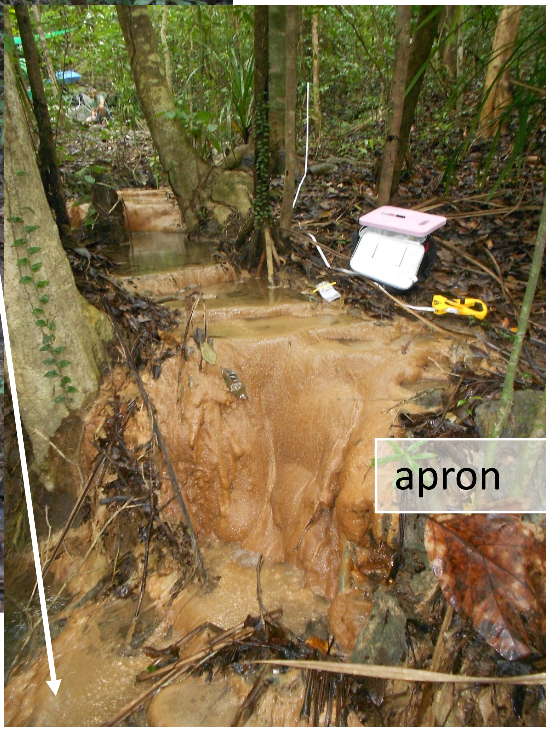

c] Manleluag Spring #2, outflow sample locations.
Asterisk marks locations of fluid sampling.

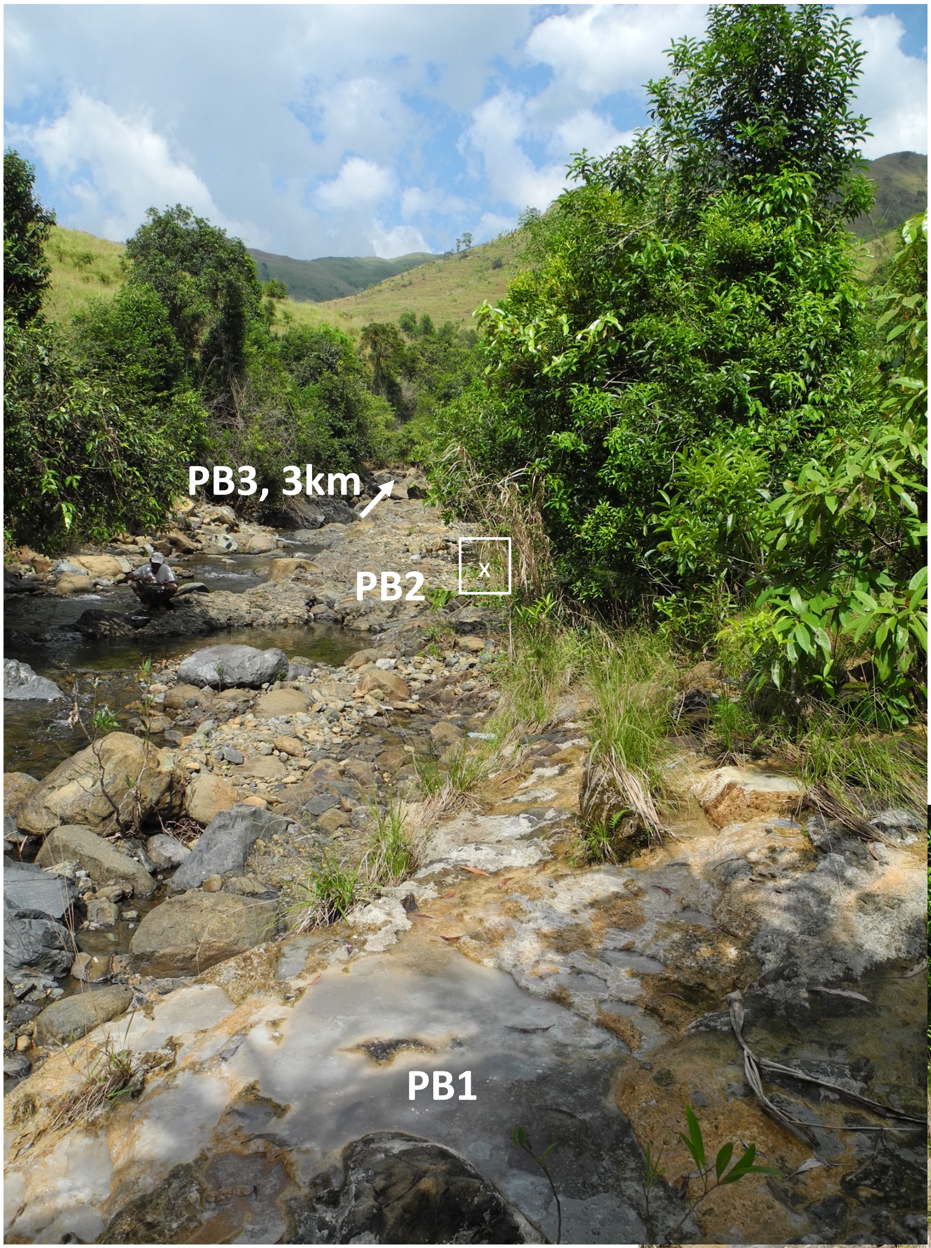

d] Poon Bato region, and pool 'PB1.' Site is natural, low flow, on the bank of the Poon Bato River. PB1 is a capped pool (note calcite cap).
Images are from 2012. In 2013 (wet season), most locations were underwater from the rising river.

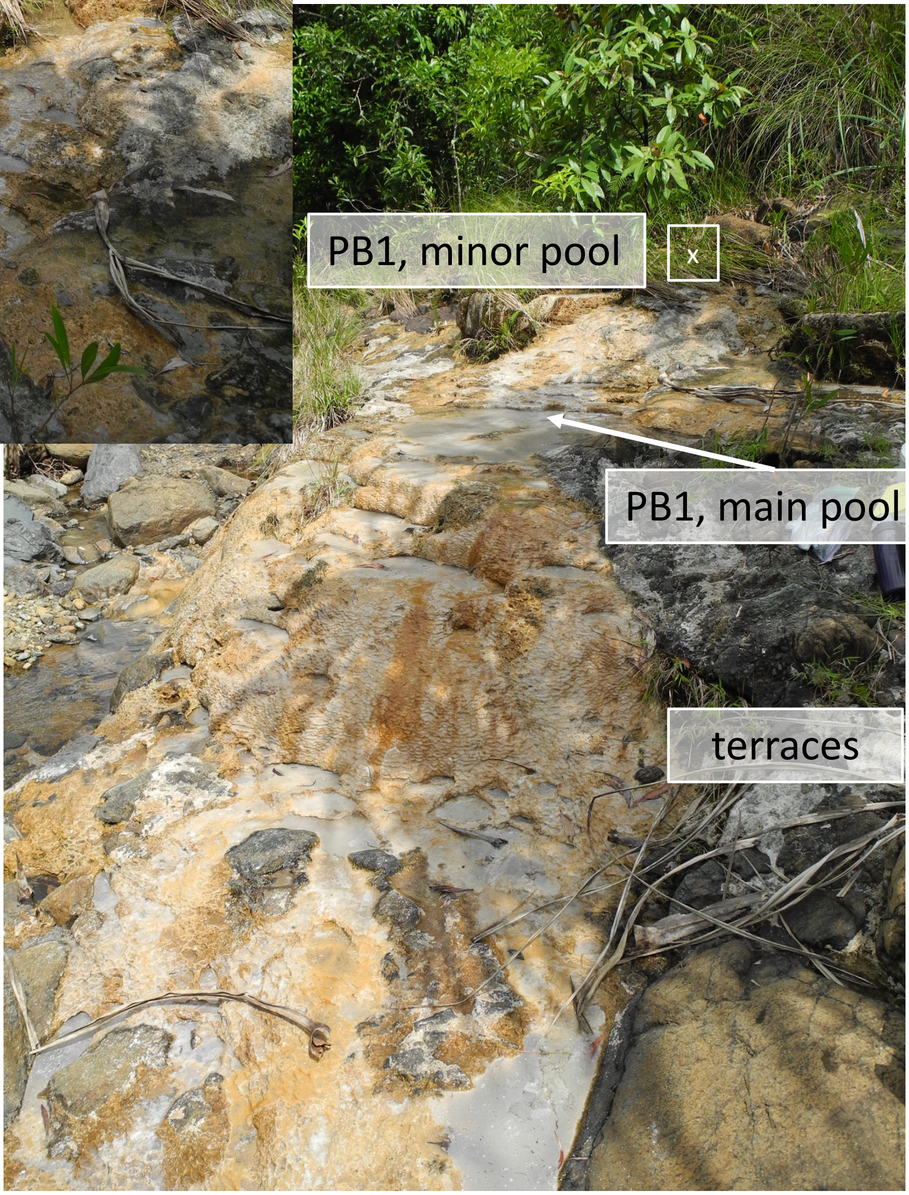

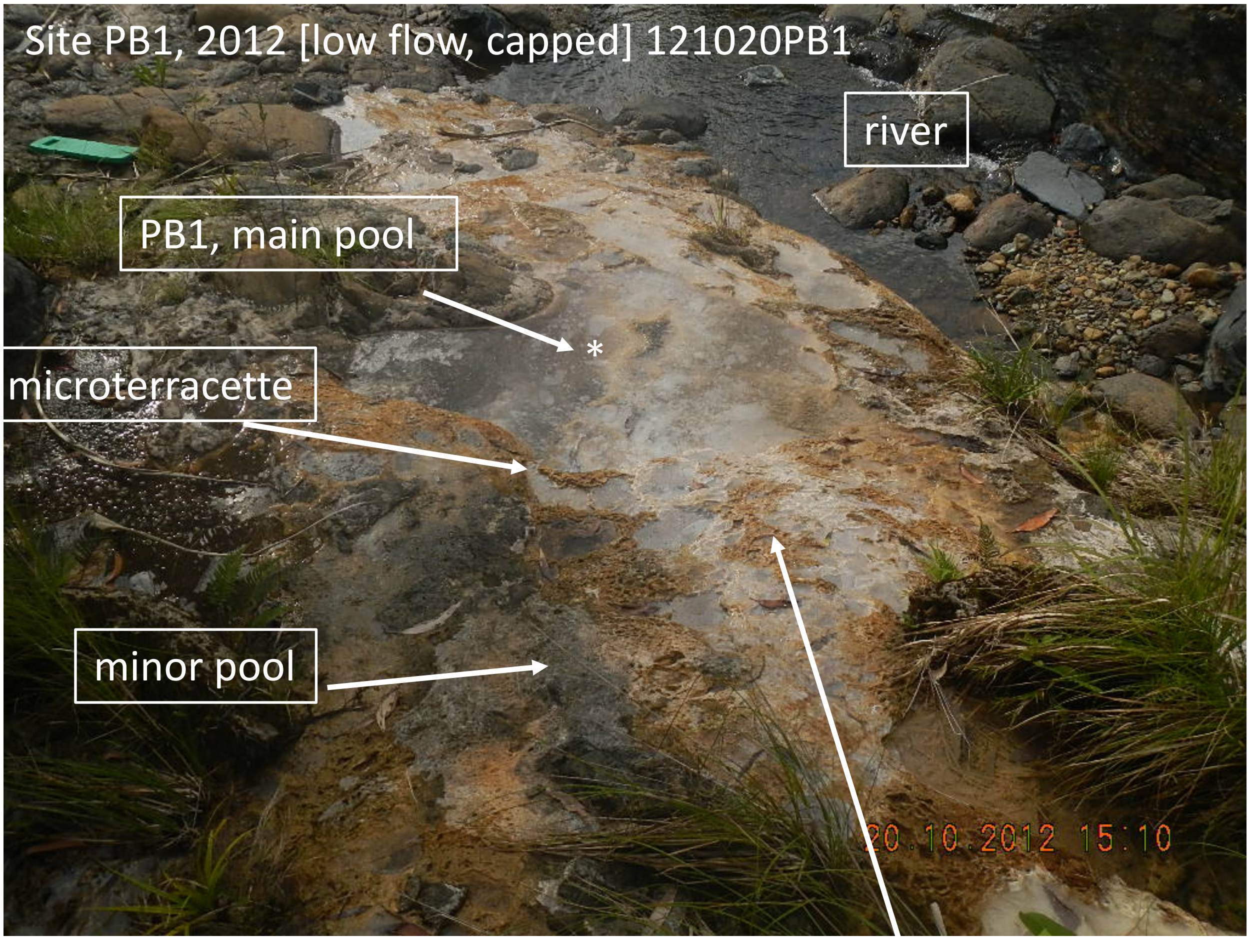

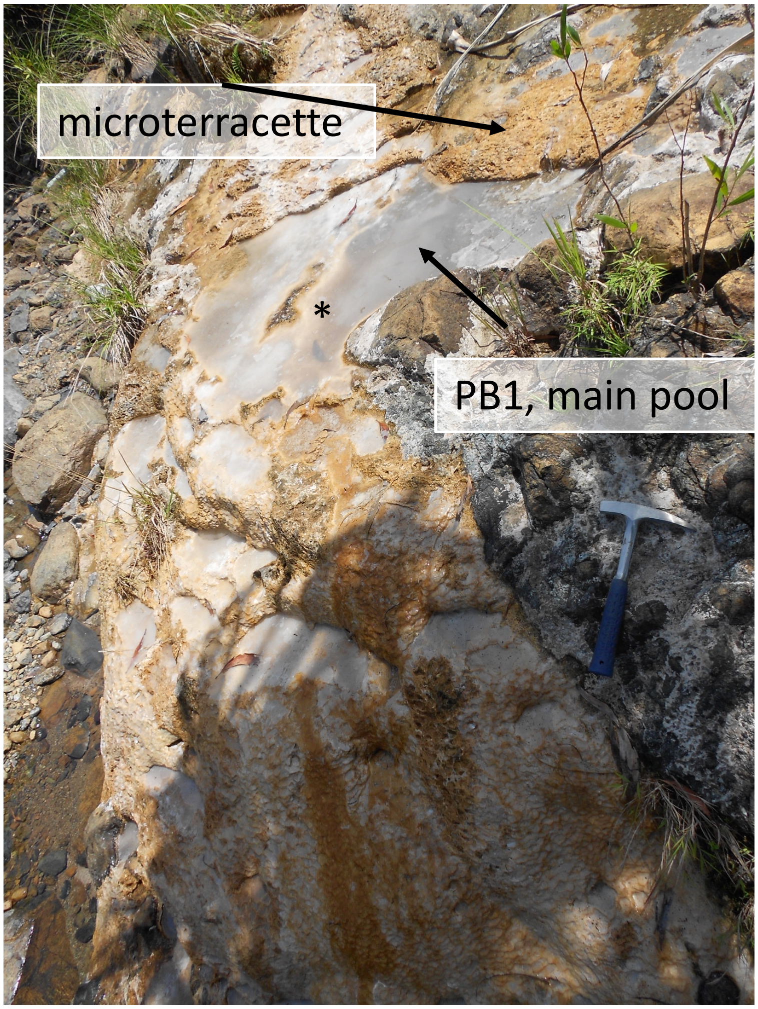

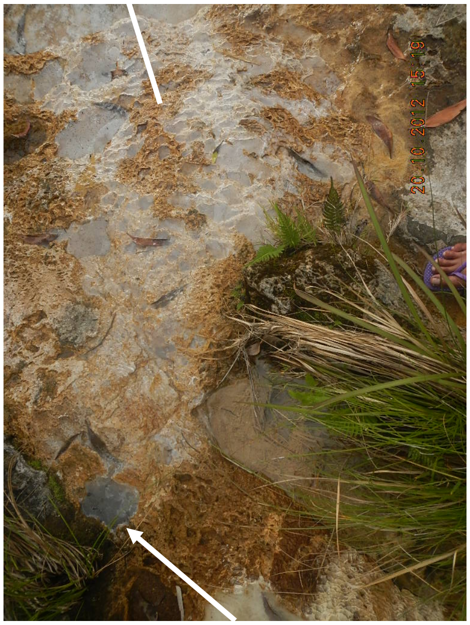

~10cm source, by minor pool

e] Poon Bato region, and pool 'PB1.' Images are from 2012. Asterisk marks location of fluid sampling .

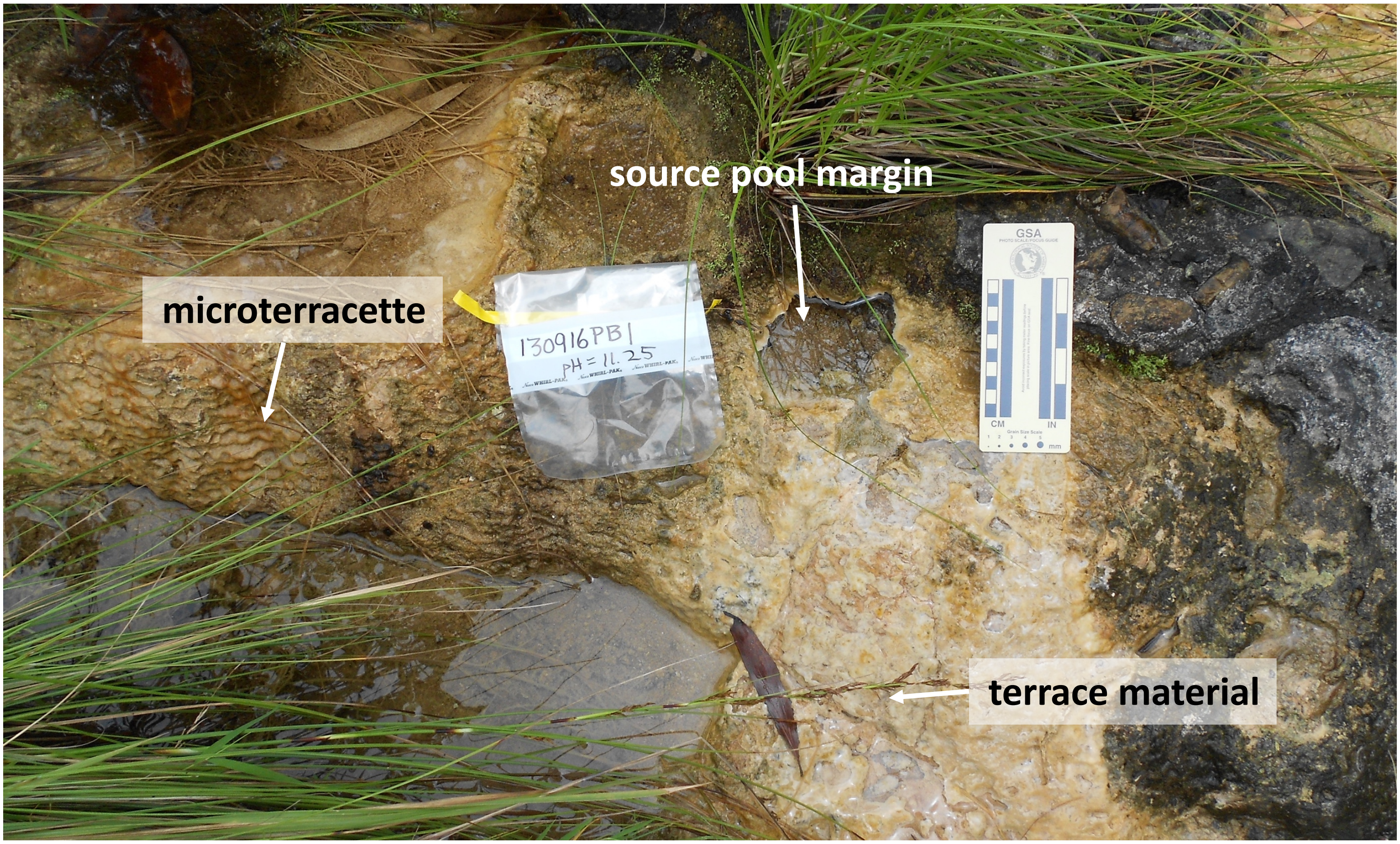

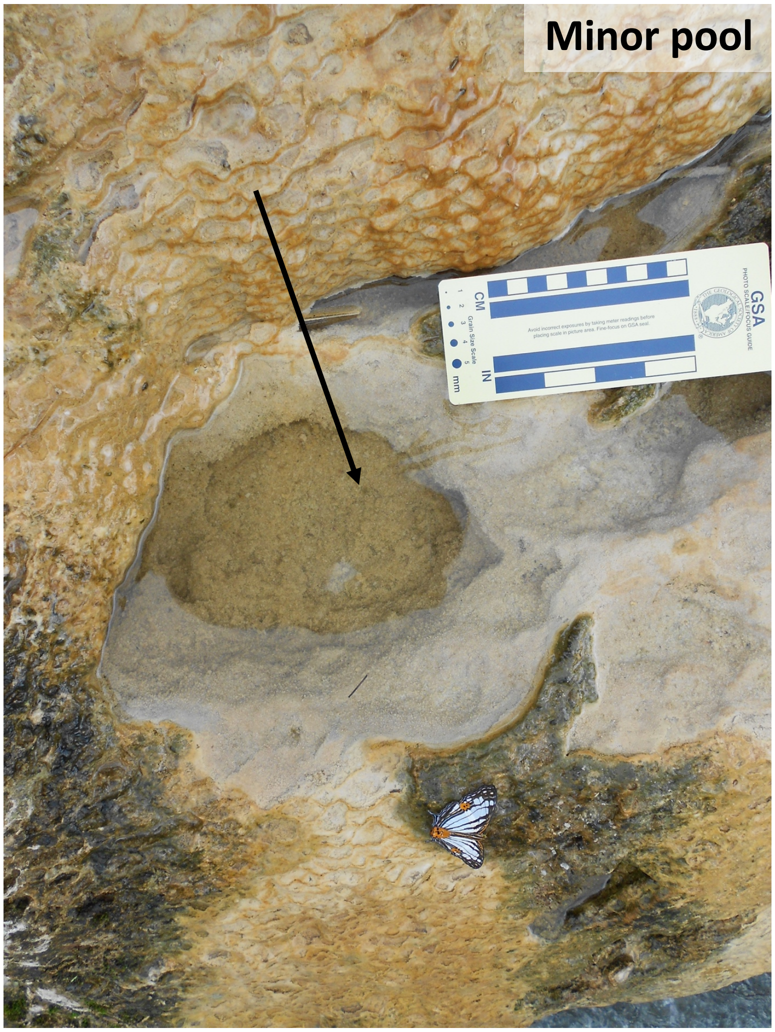

f] This upper area of PB1 was all that remained of the site in 2013 - high rains/river covered the rest.

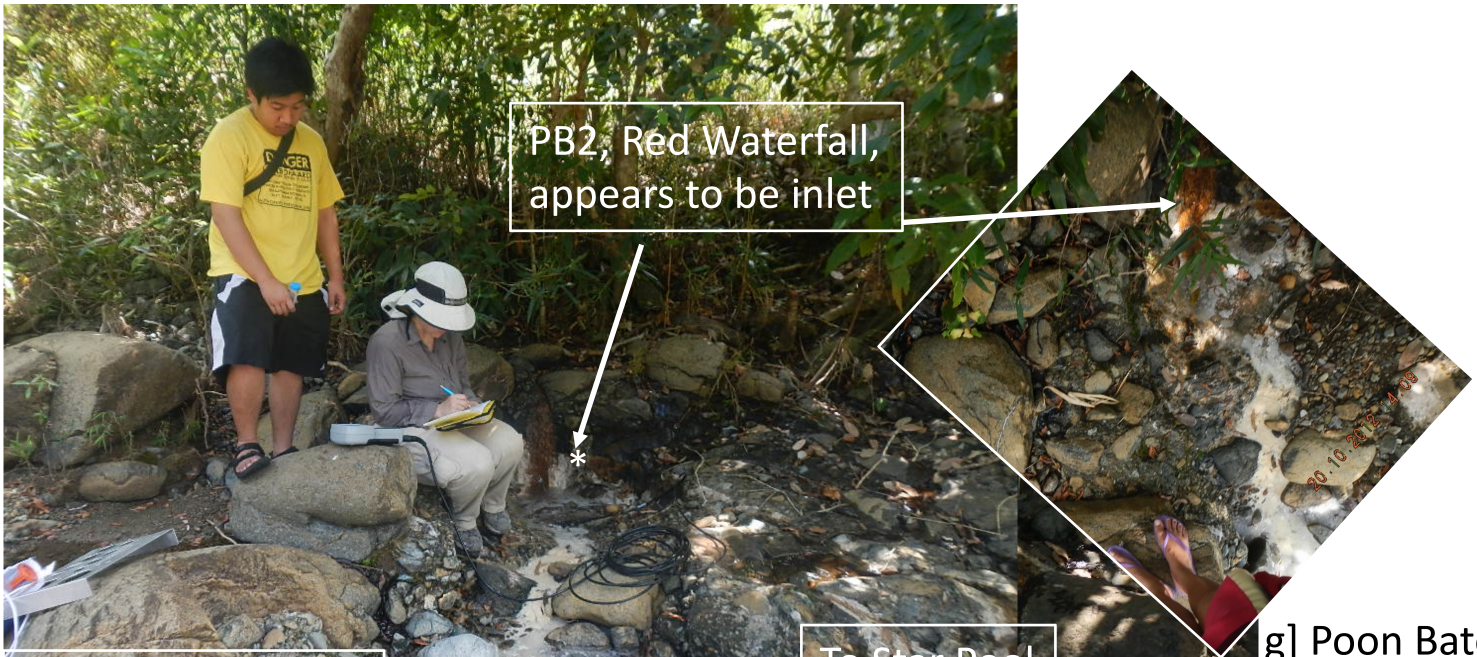

g] Poon Bato region, and pool 'PB2.' PB2 is an uncapped pool. Images are from 2012. Site was underwater in 2013. Asterisks show fluid sampling.

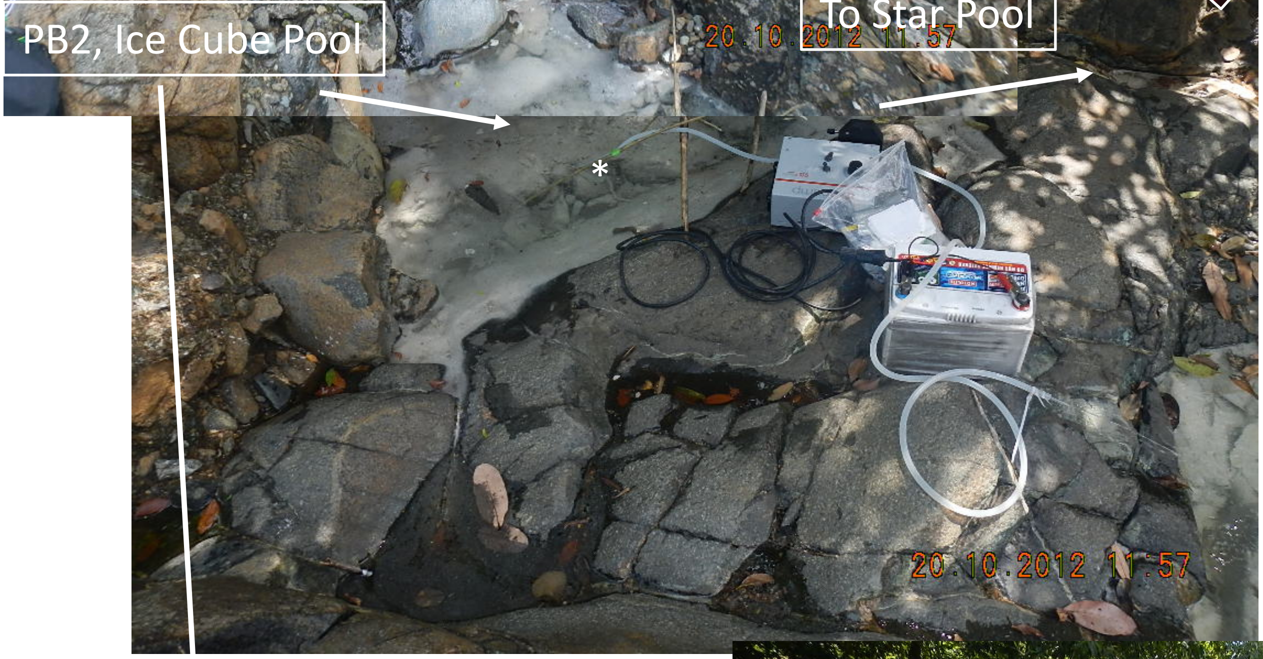

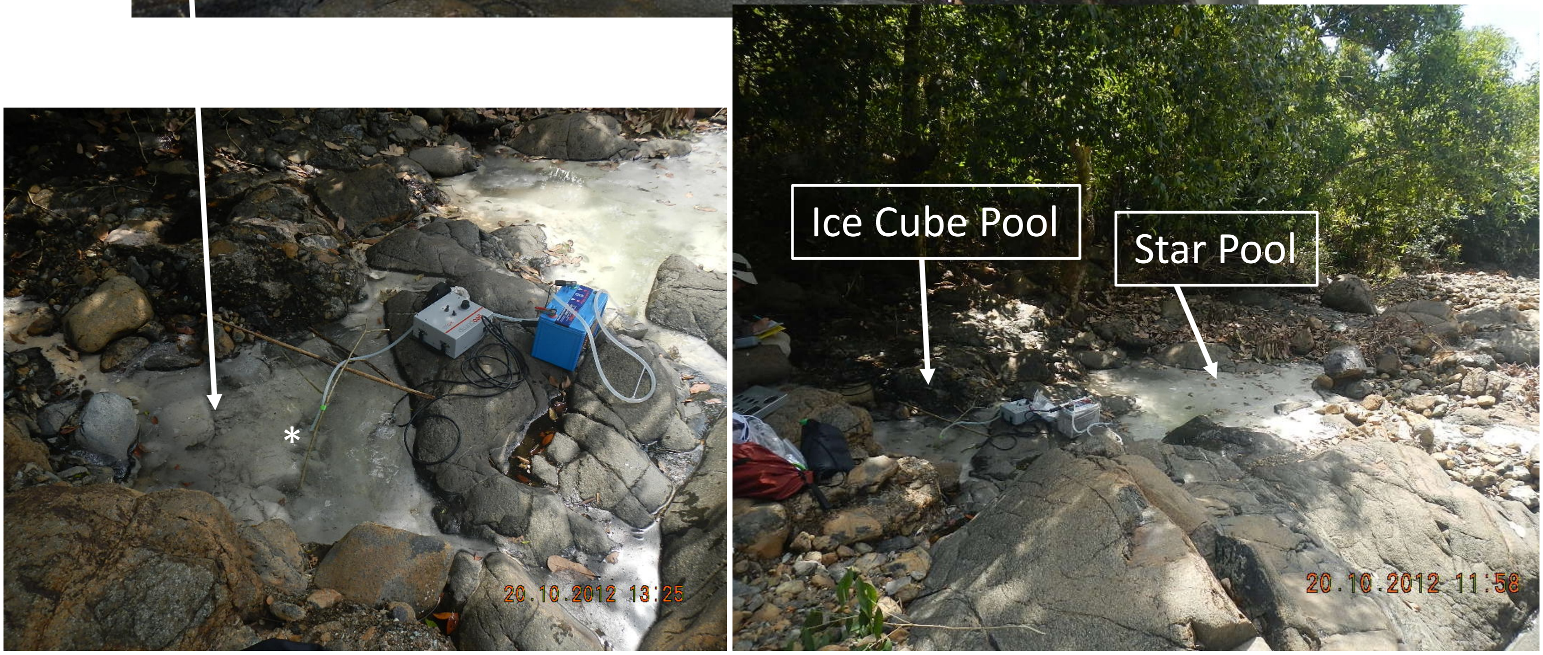

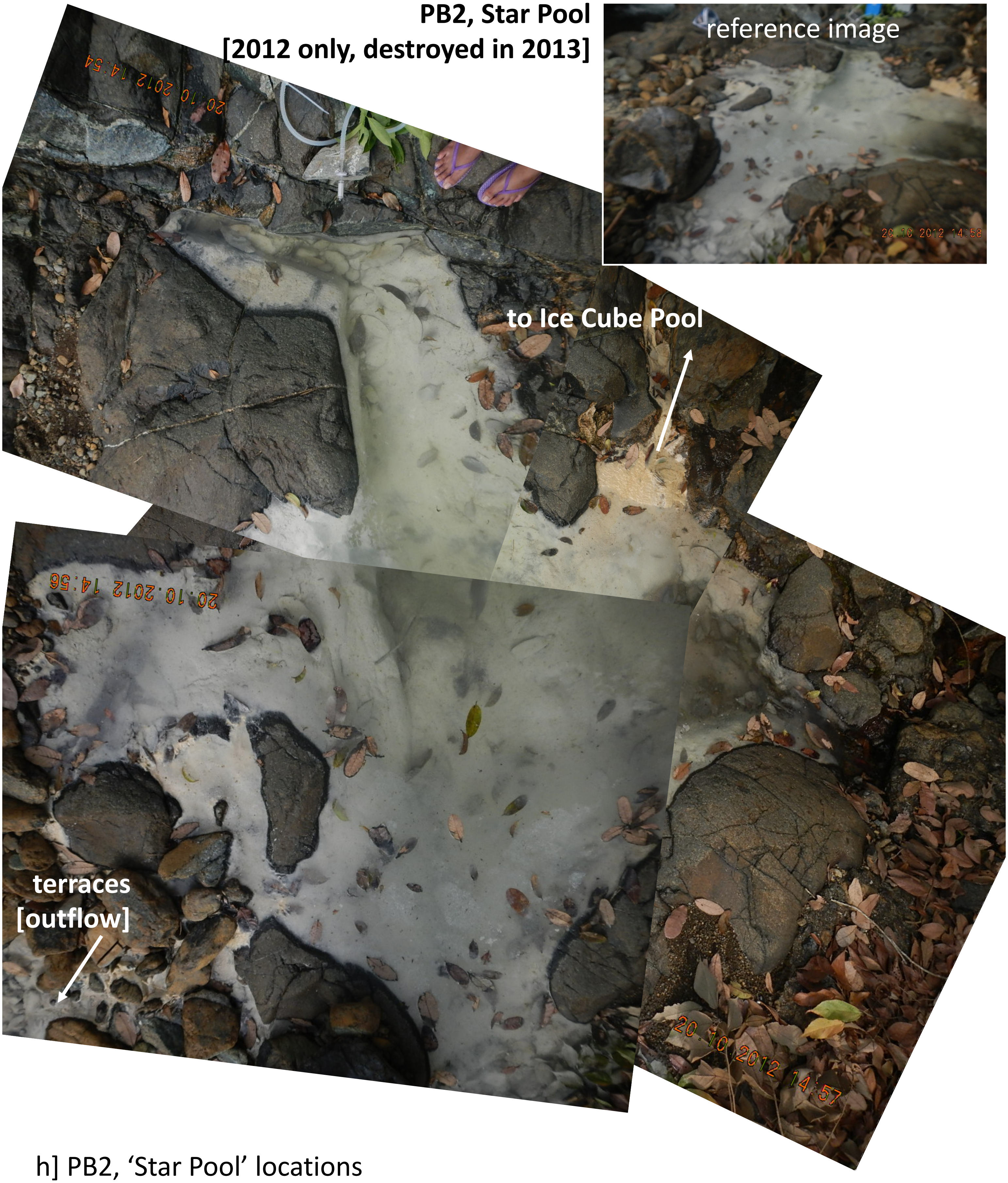

h] PB2, 'Star Pool' locations

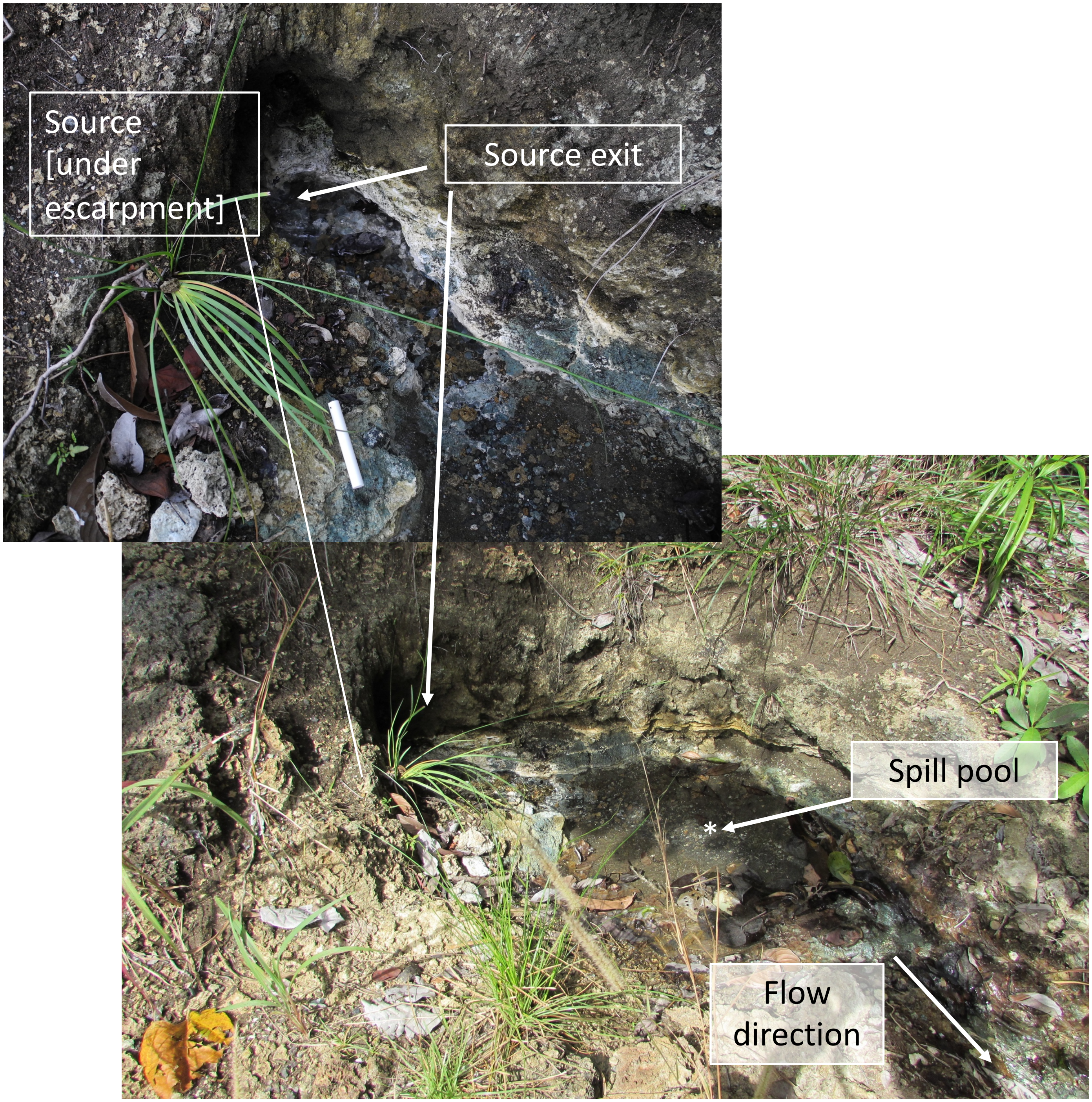

i] Governor's spring (GS) source pool locations. GS is a high flow, natural spring situated on the flank of a hillside, with an extensive runoff channel. Site was sampled in 2017. White, 5cm scale bar is shown in upper image. Asterisk marks location of fluid sampling (sample at source taken under the escarpment).

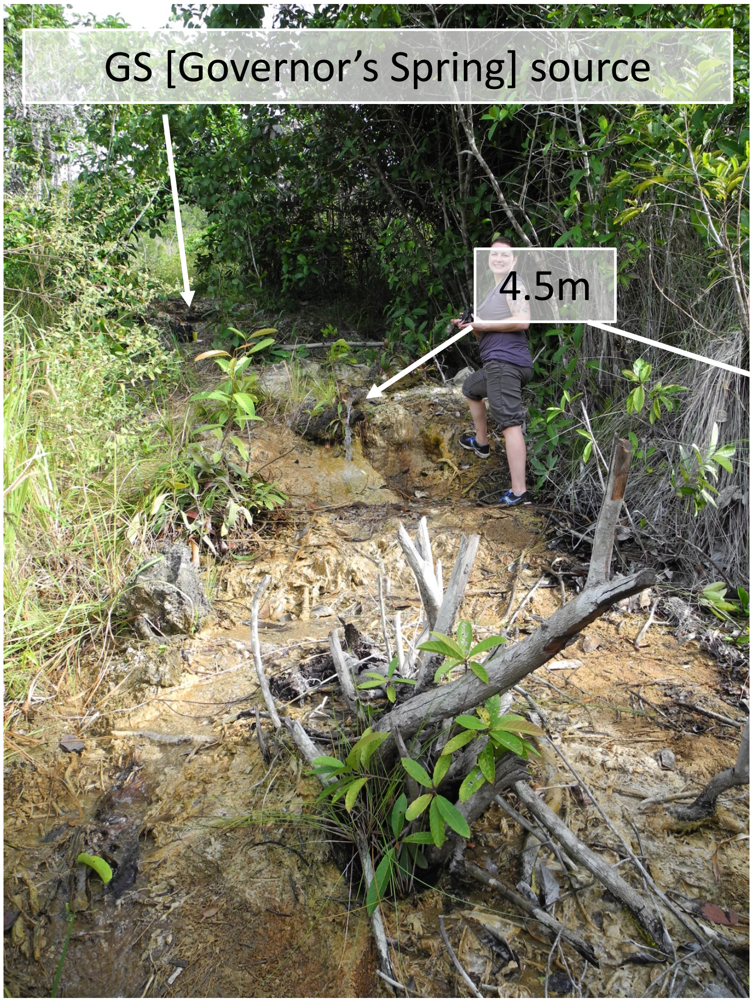

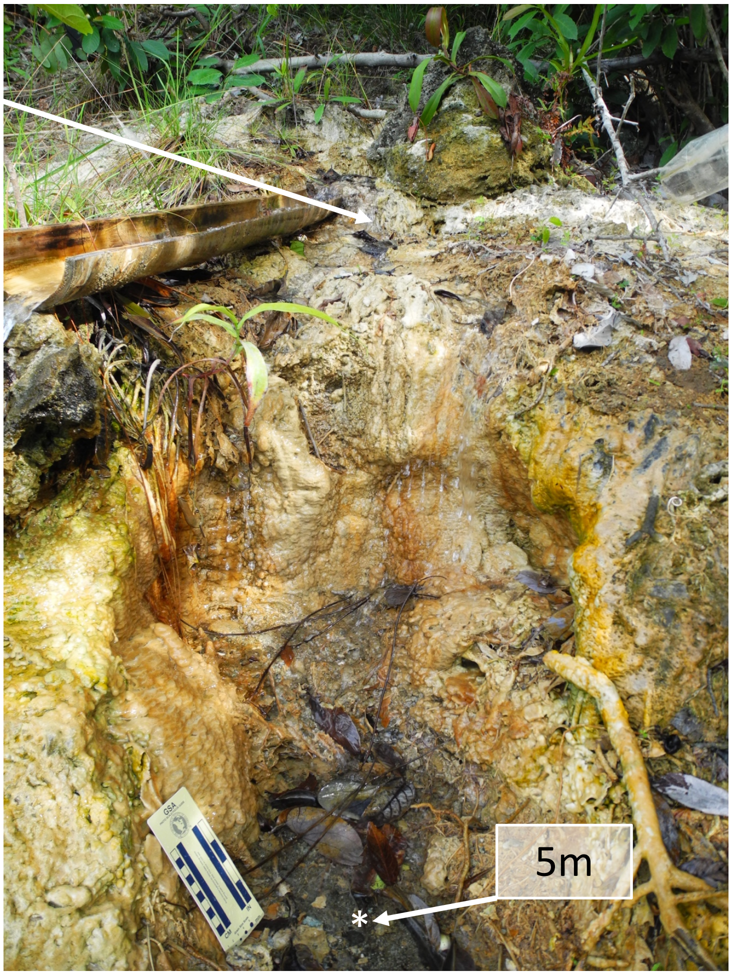

j] Governor's spring (GS) outflow locations. Site was sampled in 2017. Asterisk marks locations of fluid sampling.

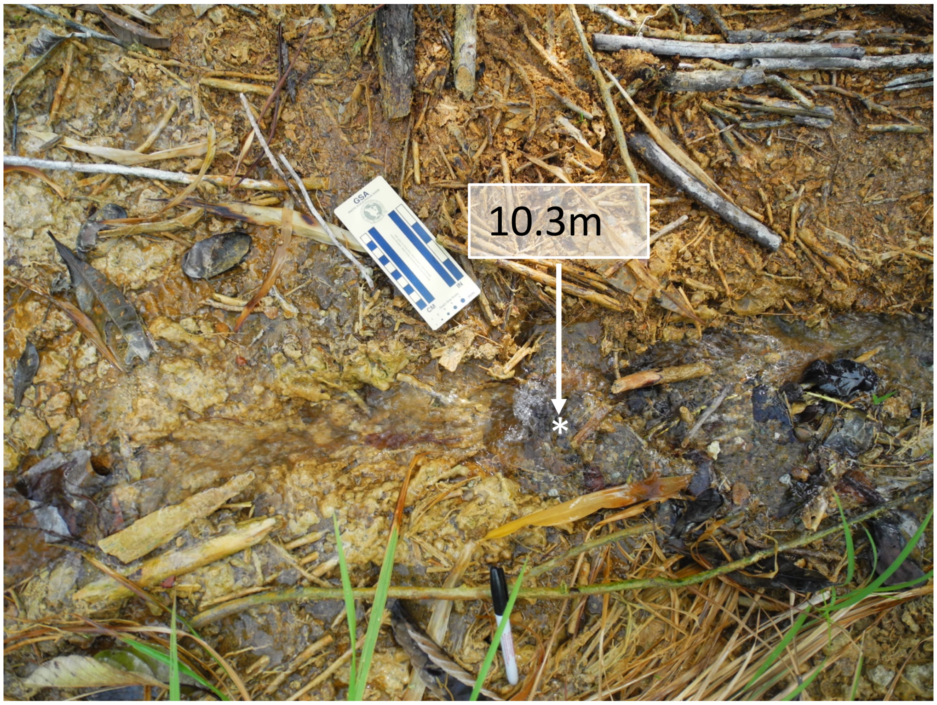

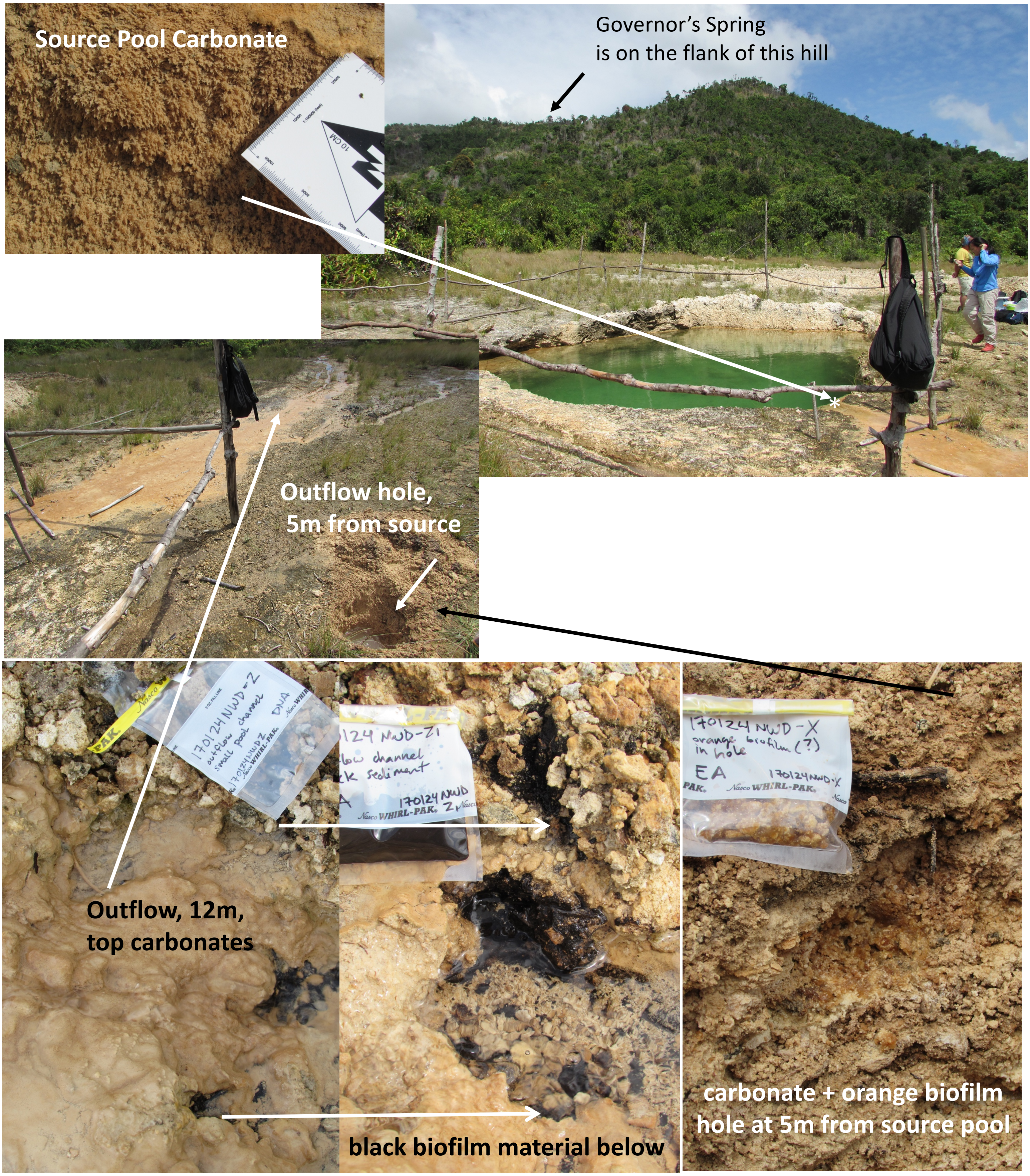

k] North West Dugout locations (NWD). Site is a man made pit, in-filled with groundwater. The surface of the pool and runoff are high flow (bottom of the pool may be low flow). Site was sampled in 2017. Arrows connect images showing the same positions. Asterisk marks location of fluid sampling.

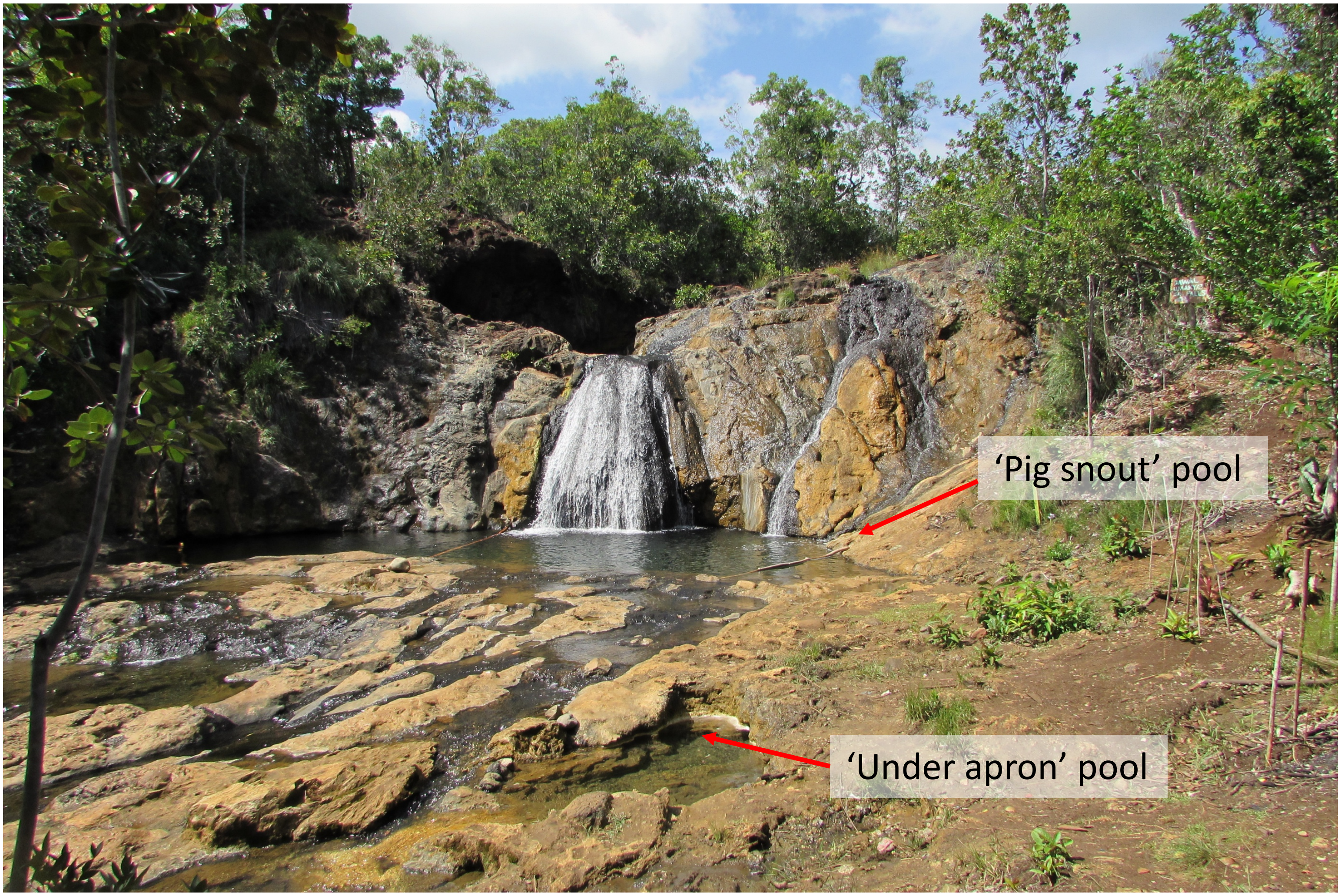

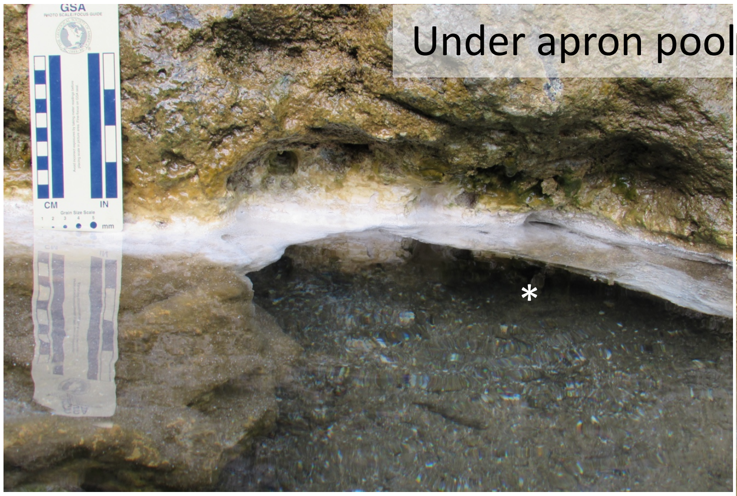

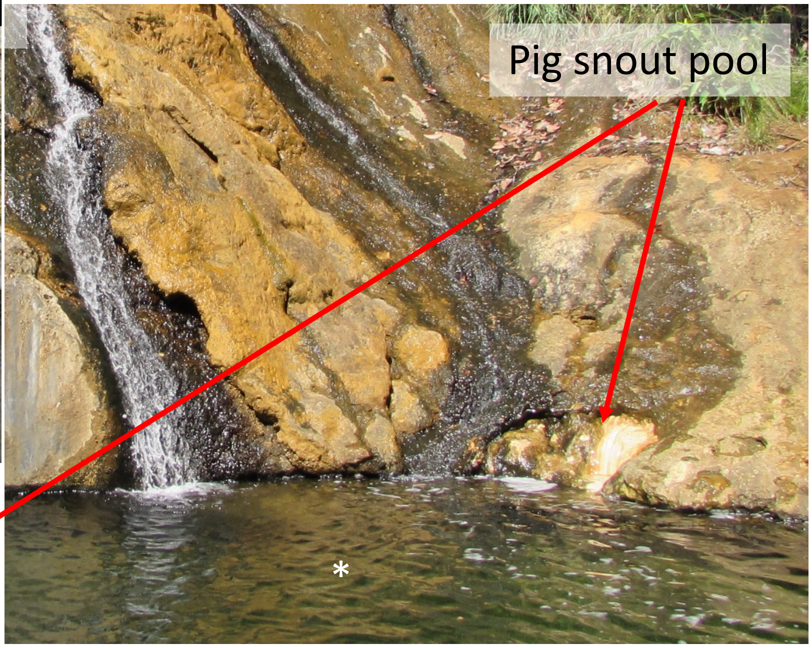

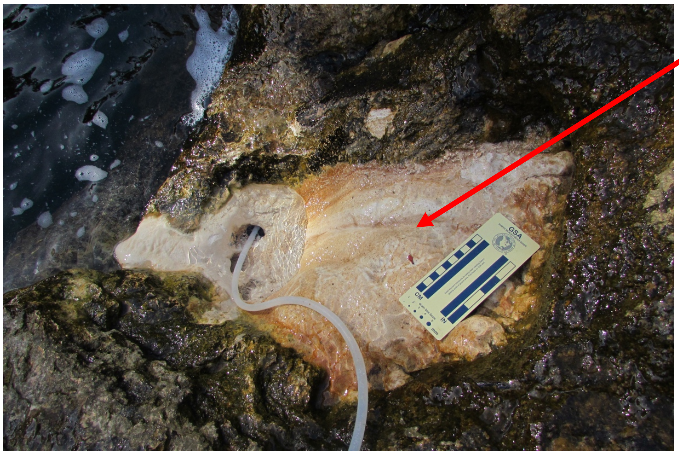

l] Pinaduguan Falls (PF) sample locations. Site is natural, low flow, uncapped and was sampled in 2017. Asterisk marks locations of fluid sampling (tubing shows location for Pig snout pool).

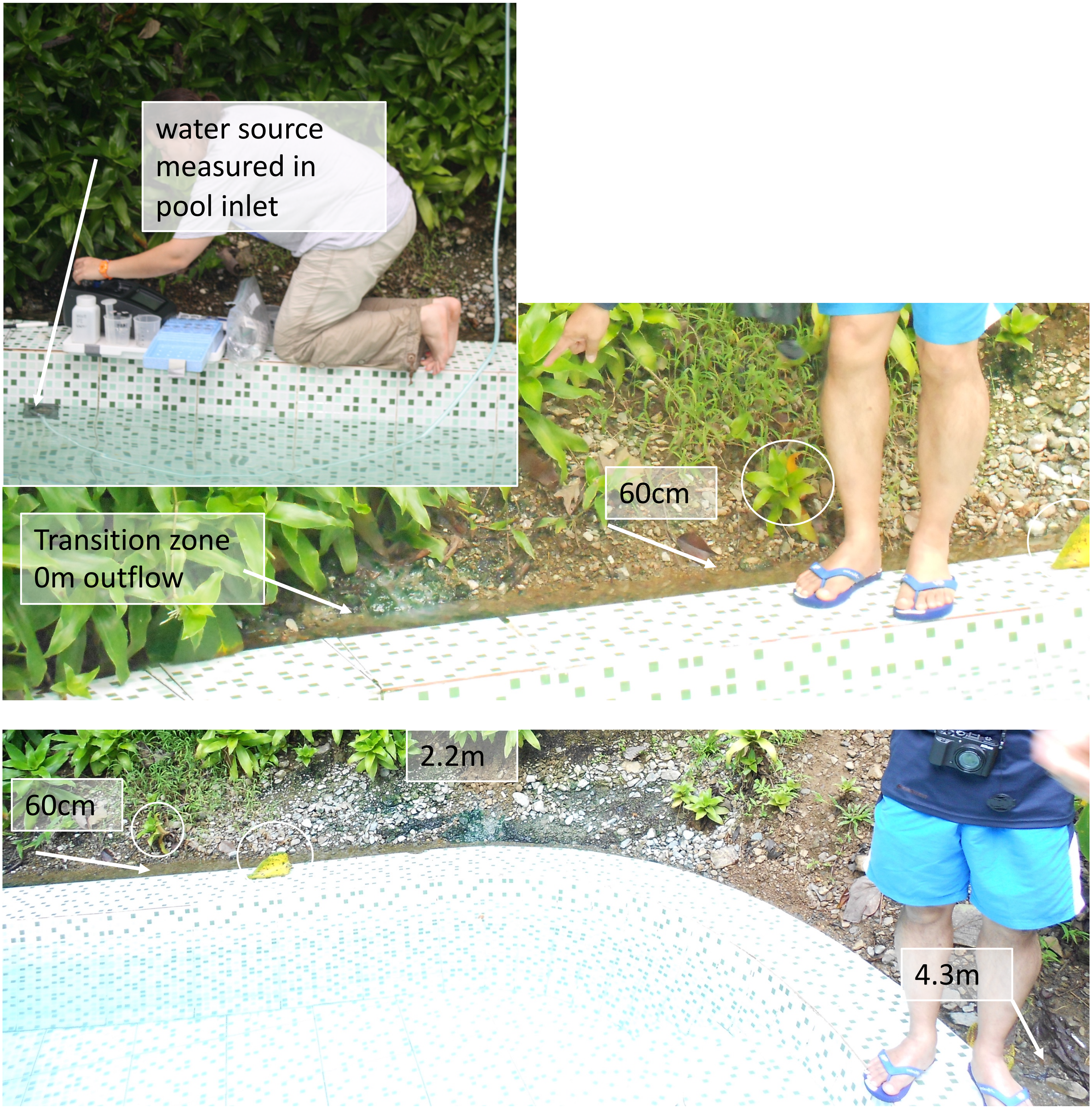

m] Mainit Falls location (MF). Small natural seep at the side of a bathing pool complex. Bathing pools are fed by the source waters. Fluid runs along the lip of the bathing pool, with obvious biofilms. Area was sampled in 2012, dry season.

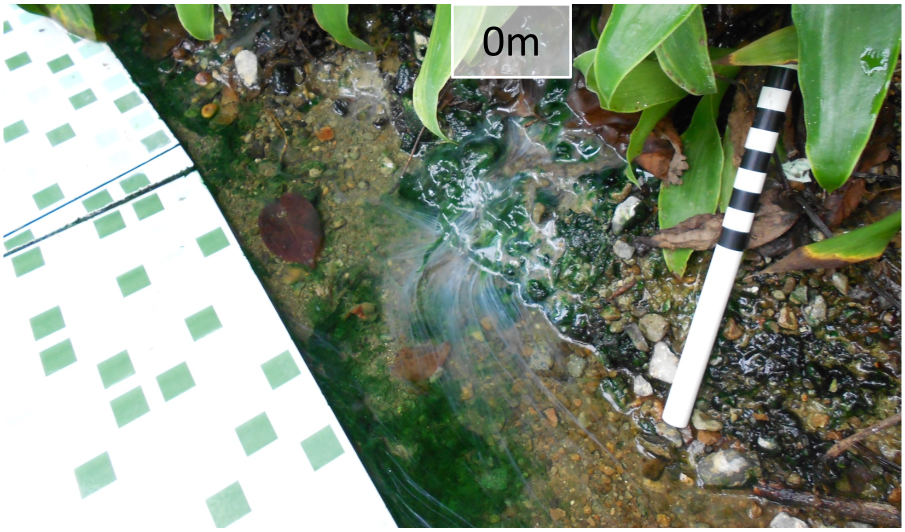

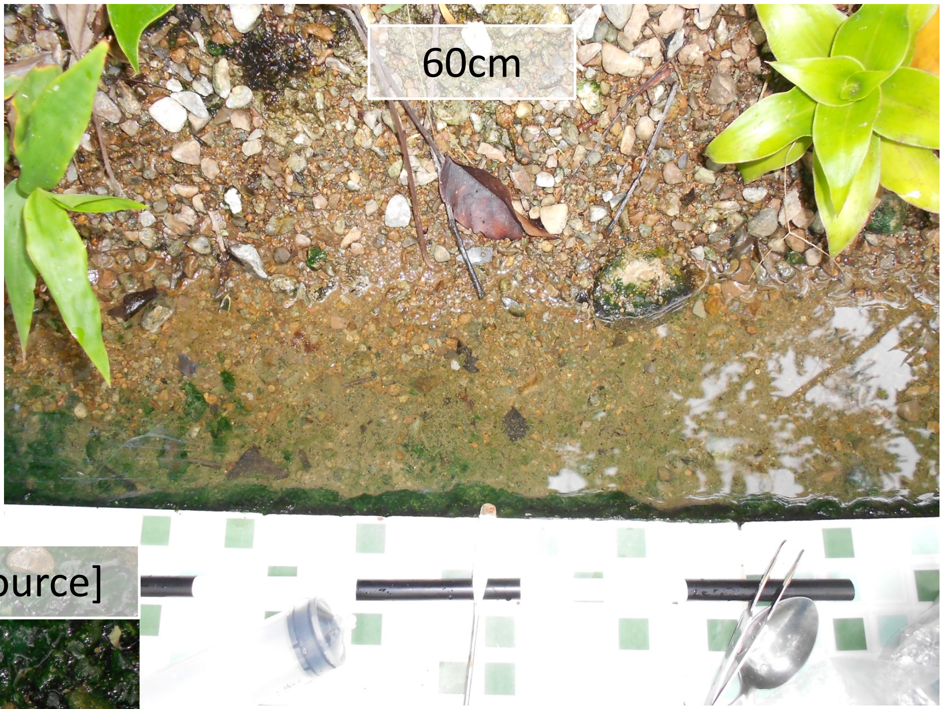

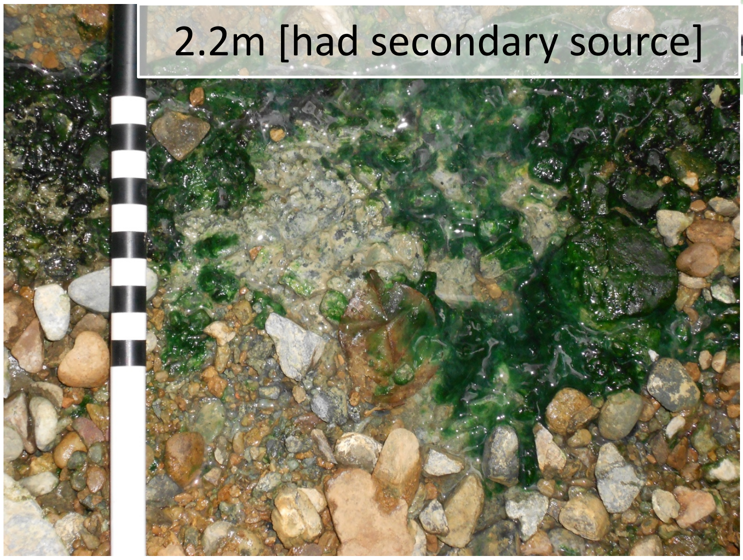

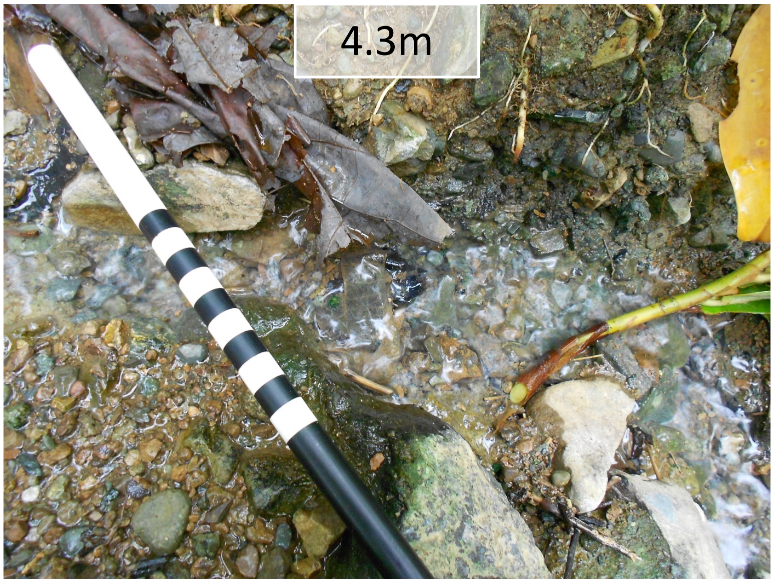

n] Mainit Falls location. Close up images of outflow sampling locations.
