# Supplementary material for: The Effect of a Tropical Climate on Available Nutrient Resources to Springs in Ophiolite-Hosted, Deep Biosphere Ecosystems in the Philippines
Source: Front Microbiol. 2019 May 1;10:761. doi: 10.3389/fmicb.2019.00761 (PMC6504838; doi:10.3389/fmicb.2019.00761)
Supplement: Supplementary file 5 [file Image_2.pdf]

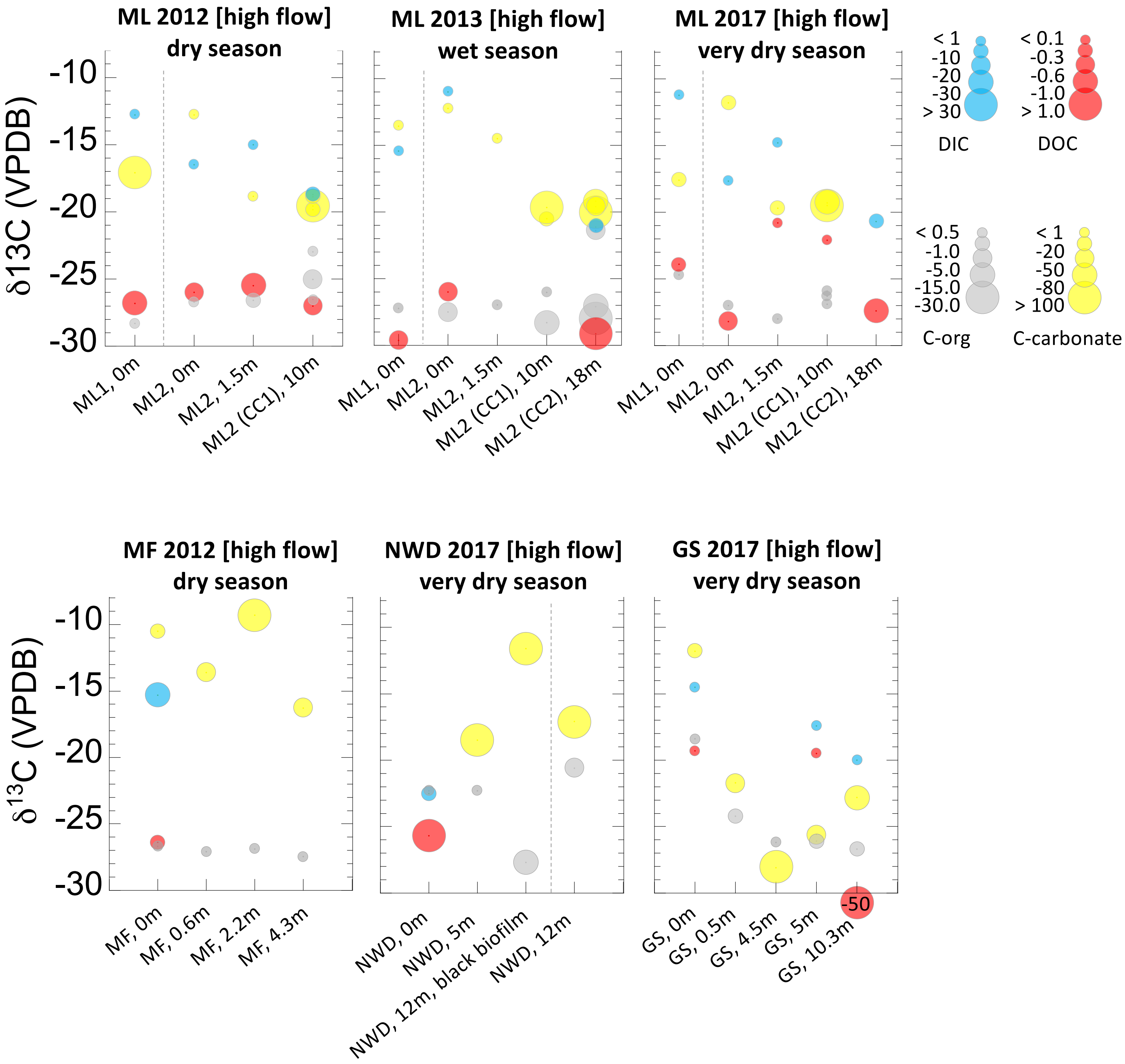


**Supplemental Figure 2a.** Ranges of concentrations and $\delta^{13}$C isotopic composition of dissolved carbon (DIC, DOC), and solid carbon (C-org, C-carbonate) in the high flow systems. Concentration ranges are given by the size of the circle for each value (key at right). Refer to table 1 for full sample names and distances along the outflow. Data are also separated by seasonal sampling, and sample names correspond to the names in the sample location pictures (Fig. 1, Fig S1) and table 1. Dashed lines separate discrete samples within a season.

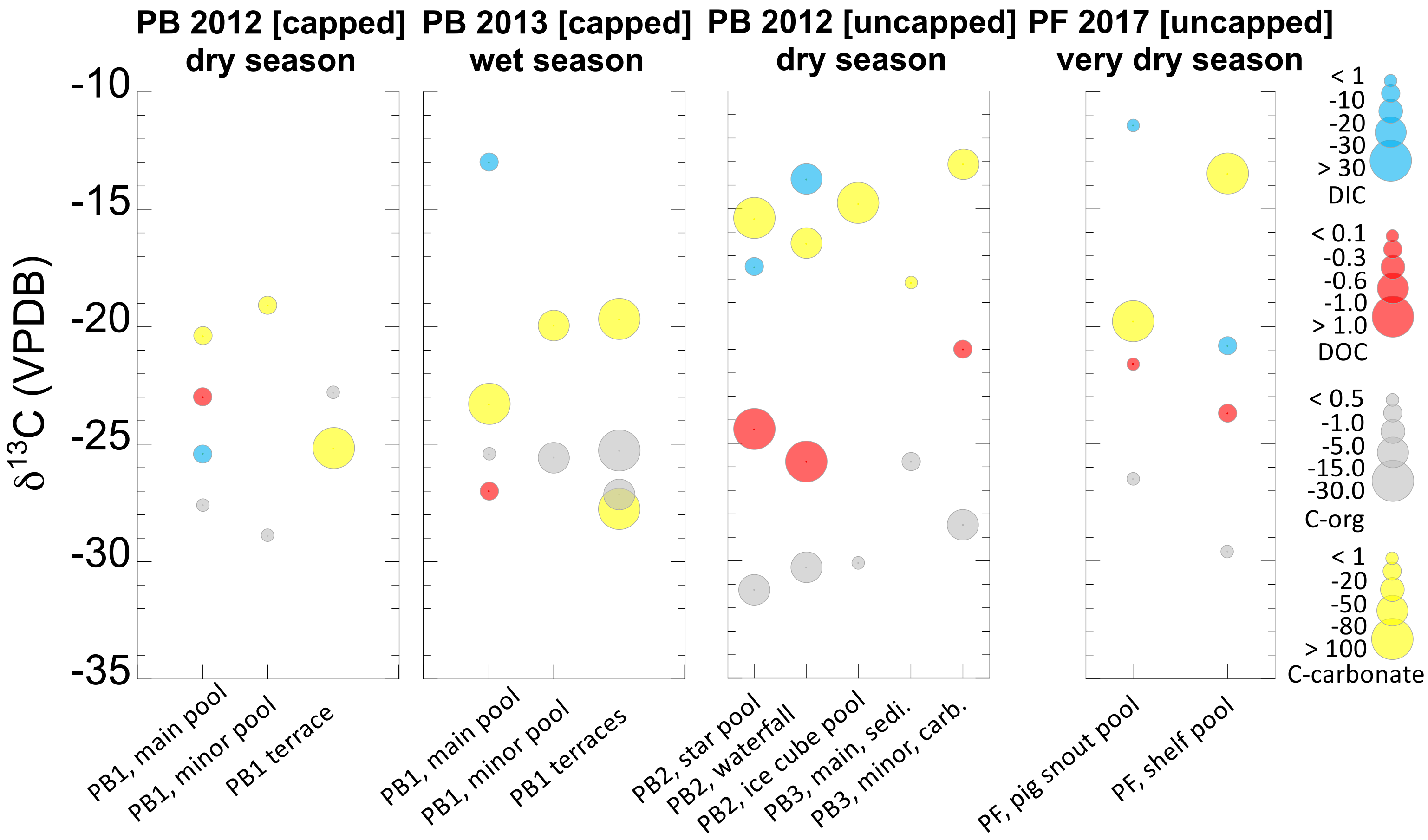


**Supplemental Figure 2b.** Ranges of concentrations and $\delta^{13}C$ isotopic composition of dissolved carbon (DIC, DOC), and solid carbon (C-org, C-carbonate) in the low flow systems. Concentration ranges are given by the size of the circle for each value (key at right). Refer to table 1 for full sample names and distances along the outflow. Data are also separated by seasonal sampling, and sample names correspond to the names in the sample location pictures (Fig. 1, Fig S1) and table 1. Dashed lines separate discrete samples within a season.
