# Supplementary figures and images for: The Effect of a Tropical Climate on Available Nutrient Resources to Springs in Ophiolite-Hosted, Deep Biosphere Ecosystems in the Philippines
Source: Front Microbiol. 2019 May 1;10:761. doi: 10.3389/fmicb.2019.00761 (PMC6504838; doi:10.3389/fmicb.2019.00761)

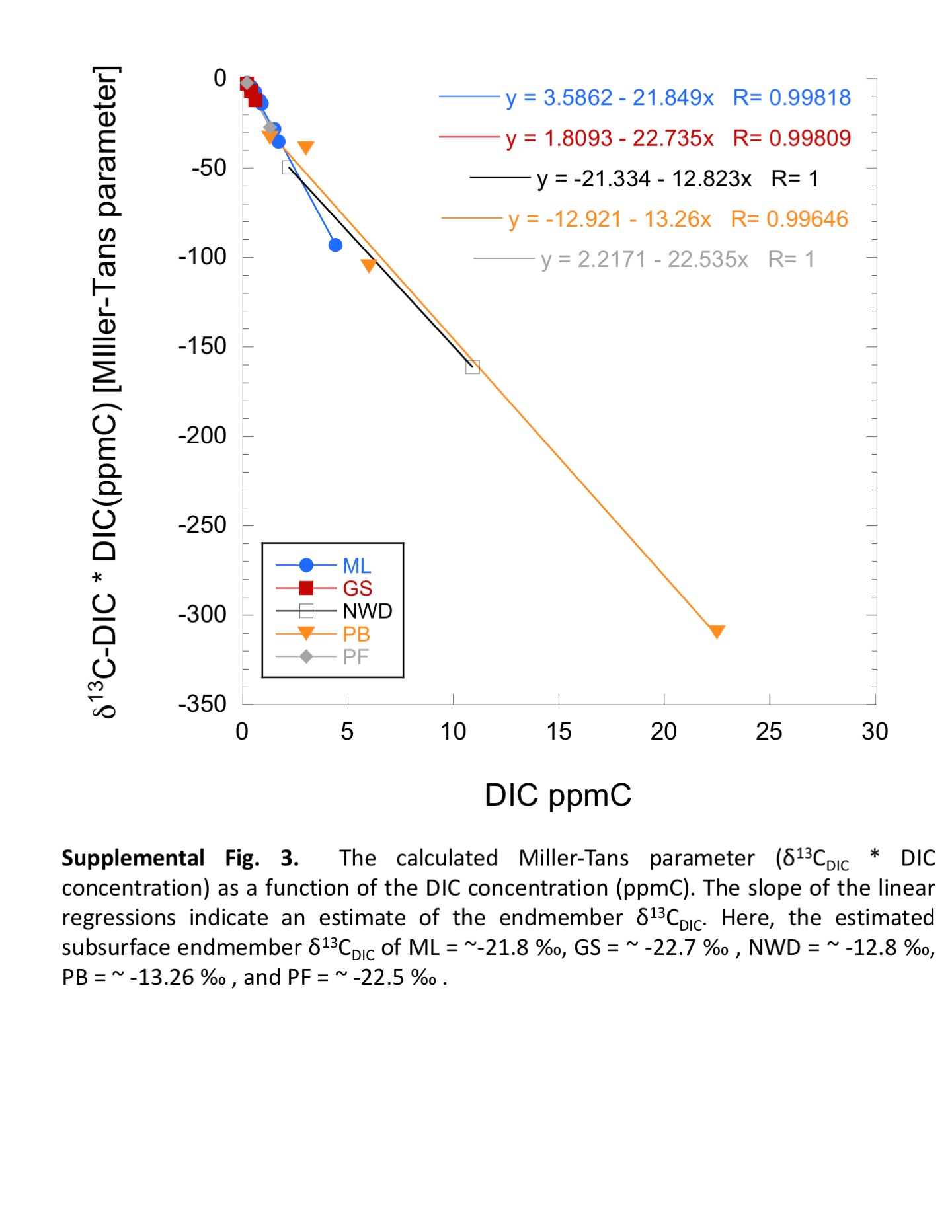

Supplement: Supplementary file 6 [file Image_3.JPEG]

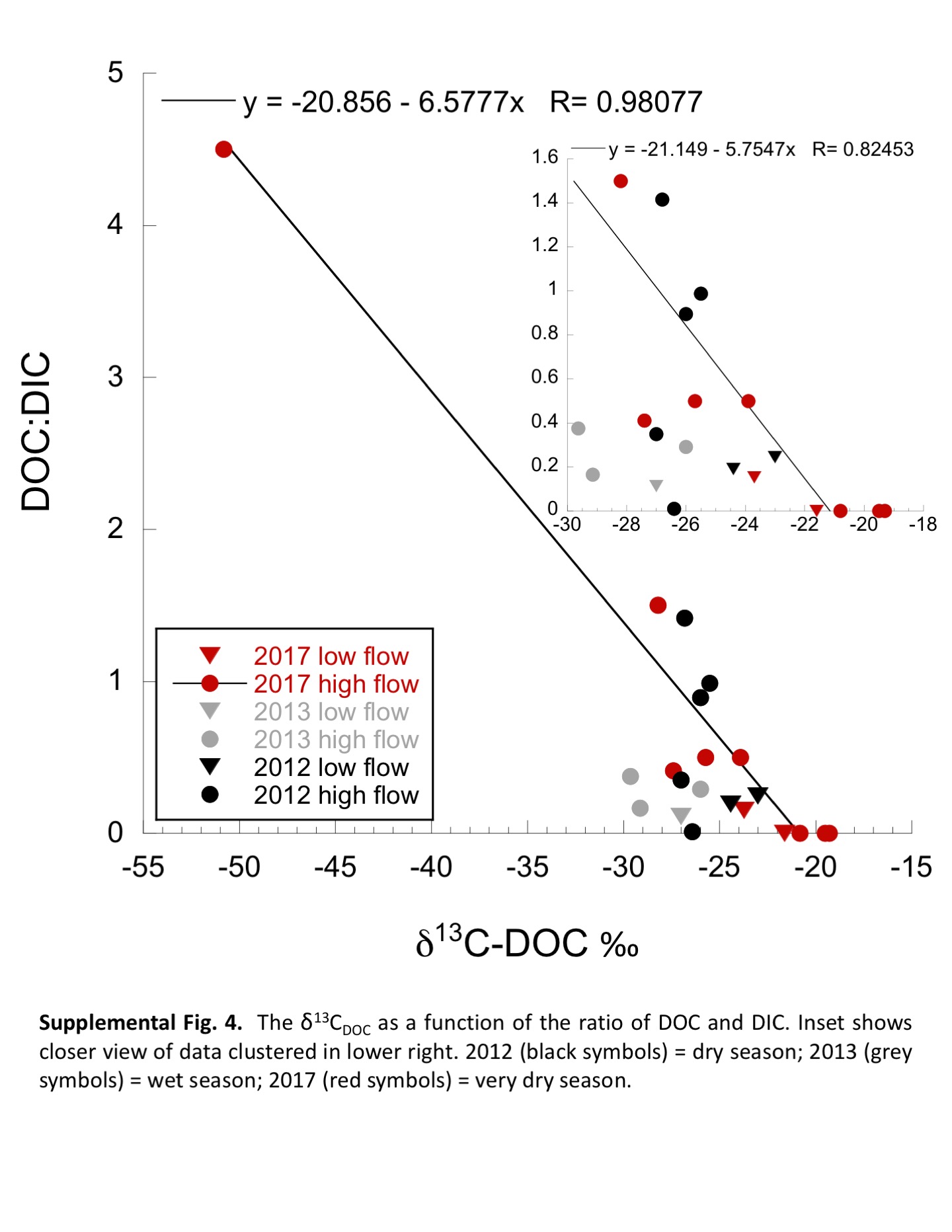

Supplement: Supplementary file 7 [file Image_4.JPEG]
